# Enamel ribbons, surface nodules, and octacalcium phosphate in C57BL/6 *Amelx* ^*−/−*^ mice and *Amelx* ^*+/−*^ lyonization

**DOI:** 10.1002/mgg3.252

**Published:** 2016-10-05

**Authors:** Yuanyuan Hu, Charles E. Smith, Zhonghou Cai, Lorenza A.‐J. Donnelly, Jie Yang, Jan C.‐C. Hu, James P. Simmer

**Affiliations:** ^1^ Department of Biologic and Materials SciencesUniversity of Michigan School of Dentistry1210Eisenhower PlaceAnn ArborMichigan48108; ^2^ Facility for Electron Microscopy ResearchDepartment of Anatomy and Cell BiologyFaculty of DentistryMcGill UniversityMontrealQuebecH3A 2B2Canada; ^3^ Advanced Photon SourceArgonne National Laboratory9700 S. Cass Ave Building 431‐B005ArgonneIllinois60439; ^4^ Department of Pediatric DentistrySchool and Hospital of StomatologyPeking University22 South AvenueZhongguancun Haidian DistrictBeijing100081China

**Keywords:** Ameloblast, amelogenesis imperfecta, amelogenin, amorphous calcium phosphate, enamel, incisor, molar, octacalcium phosphate

## Abstract

**Background:**

Amelogenin is required for normal enamel formation and is the most abundant protein in developing enamel.

**Methods:**

*Amelx* ^+/+^, *Amelx* ^+/*−*^, and *Amelx* ^*−*/*−*^ molars and incisors from C57BL/6 mice were characterized using RT‐PCR, Western blotting, dissecting and light microscopy, immunohistochemistry (IHC), transmission electron microscopy (TEM), scanning electron microscopy (SEM), backscattered SEM (bSEM), nanohardness testing, and X‐ray diffraction.

**Results:**

No amelogenin protein was detected by Western blot analyses of enamel extracts from *Amelx* ^*−*/*−*^ mice. *Amelx* ^*−*/*−*^ incisor enamel averaged 20.3 ± 3.3 *μ*m in thickness, or only 1/6th that of the wild type (122.3 ± 7.9 *μ*m). *Amelx* ^*−*/*−*^ incisor enamel nanohardness was 1.6 Gpa, less than half that of wild‐type enamel (3.6 Gpa). *Amelx* ^+/*−*^ incisors and molars showed vertical banding patterns unique to each tooth. IHC detected no amelogenin in *Amelx* ^*−*/*−*^ enamel and varied levels of amelogenin in *Amelx* ^+/*−*^ incisors, which correlated positively with enamel thickness, strongly supporting lyonization as the cause of the variations in enamel thickness. TEM analyses showed characteristic mineral ribbons in *Amelx* ^+/+^ and *Amelx* ^*−*/*−*^ enamel extending from mineralized dentin collagen to the ameloblast. The *Amelx* ^*−*/*−*^ enamel ribbons were not well separated by matrix and appeared to fuse together, forming plates. X‐ray diffraction determined that the predominant mineral in *Amelx* ^*−*/*−*^ enamel is octacalcium phosphate (not calcium hydroxyapatite). *Amelx* ^*−*/*−*^ ameloblasts were similar to wild‐type ameloblasts except no Tomes’ processes extended into the thin enamel. *Amelx* ^*−*/*−*^ and *Amelx* ^+/*−*^ molars both showed calcified nodules on their occlusal surfaces. Histology of D5 and D11 developing molars showed nodules forming during the maturation stage.

**Conclusion:**

Amelogenin forms a resorbable matrix that separates and supports, but does not shape early secretory‐stage enamel ribbons. Amelogenin may facilitate the conversion of enamel ribbons into hydroxyapatite by inhibiting the formation of octacalcium phosphate. Amelogenin is necessary for thickening the enamel layer, which helps maintain ribbon organization and development and maintenance of the Tomes’ process.

## Introduction

Amelogenin is the most abundant protein in secretory‐stage enamel and is specialized for amelogenesis (Fincham et al., 1999). In humans, there are two nonallelic amelogenin genes: on the X (*AMELX*; OMIM *300391) and Y (*AMELY*; OMIM *410000) chromosomes, although the copy on the Y chromosome is expressed at relatively low levels and is not critical for proper dental enamel formation (Lau et al. 1989; Salido et al. 1992; Lattanzi et al. 2005; Hu et al. 2012). *AMELX* is nested within the large (>400 kb) first intron of *ARHGAP6* (OMIM *300118) and is transcribed in the opposite direction (Schaefer et al. 1997). In rodents there is only a single copy of the amelogenin gene (*Amelx*), and targeted interruption of this gene in mice resulted in an amelogenesis imperfecta phenotype (Gibson et al. 2001). To date, 19 different genetic defects in *AMELX* have been reported to cause X‐linked amelogenesis imperfecta (AI) (OMIM #301200) (Lagerström et al. 1990, 1991; Aldred et al. 1992; Lench et al. 1994; Lagerstrom‐Fermer et al. 1995; Lench and Winter 1995; Collier et al. 1997; Hart et al. 2000, 2002; Kindelan et al. 2000; Ravassipour et al. 2000; Sekiguchi et al. 2001a,b; Greene et al. 2002; Kim et al. 2004; Kida et al. 2007; Chan et al. 2011; Lee et al. 2011; Wright et al. 2011; Cho et al. 2014) (Appendix S1), which occurs in the absence of any phenotype except in enamel. A telltale phenotype of X‐linked AI is that heterozygous females often exhibit vertical bands of hypoplastic enamel alternating with bands of normal or less severely affected enamel, whereas affected males exhibit a uniformly thin layer of defective enamel. The distinctive vertical banding of the enamel in heterozygous females is thought to be caused by mosaicism of ameloblast cohorts with respect to functional amelogenin expression, which in turn is secondary to random X‐chromosome inactivation earlier during development (lyonization) (Lyon 1961; Witkop 1967). Vertical banding of the enamel is also observed in focal dermal hypoplasia (OMIM #305600), an X‐linked dominant condition with male lethality that is caused by heterozygous mutations in *PORCN* (OMIM *300651) (Gysin and Itin 2015).

Amelogenin is specialized for dental enamel formation. Amelogenin is expressed by the ameloblast lineage starting just before the initial mineralization of dentin, while its expression terminates early in the maturation stage (Snead et al. 1988; Inai et al. 1991; Wurtz et al. 1996; Wakida et al. 1999; Hu et al. 2001). Amelogenin is transiently expressed by young odontoblasts, but this expression ends after the onset of dentin mineralization (Karg et al. 1997). Amelogenin is not expressed by Hertwig's Epithelial Root Sheath (Luo et al. 1991), along developing tooth roots (Hu et al. 2001), or by Epithelial Rests of Malassez either under normal conditions or following a periodontal challenge (Nishio et al. 2010). No amelogenin expressed sequence tags (EST) were identified among the 3.32 million ESTs reported for normal human tissues (Hs.654436), which did not sample developing teeth. Only one amelogenin EST was identified out of over 3.36 million ESTs (Mm.391342) characterized from mouse tissues (excluding developing molars). Inactivating *Amelx* mutations have been observed in all edentulous vertebrate genomes yet examined (including birds, turtles, and multiple mammalian species), as well as in the genomes of enamel‐less mammals (sloth, armadillo, and aardvark) (Meredith et al. 2014).

Amelogenin belongs to the secretory calcium‐binding phosphoprotein (SCPP) family of proteins that arose from the 5’ region of ancestral *Sparcl1* (SPARC‐like 1) (Kawasaki et al. 2004). Most SCPP genes (including *AMEL*) have all of their exons separated by phase 0 introns, so inclusion or deletions of exon(s) by alternative splicing do not shift the reading frame. Multiple alternatively spliced amelogenin transcripts have been identified by RT‐PCR of RNA isolated from the enamel organ epithelia of developing teeth from many mammalian species (Gibson et al. 1991; Lau et al. 1992; Salido et al. 1992; Hu et al. 1996; Ryu et al. 1998) and even from an amphibian (Wang et al. 2013). When alternative splicing causes the inclusion of a novel sequence, such as Exon 4 (Simmer et al. 1994a), or Exons 8 and 9 (Li et al. 1998; Bartlett et al. 2006) in rodents, antibodies have identified the amelogenin variants translated from these transcripts. However, the functional importance (if any) of amelogenins translated from alternatively spliced transcripts remains unknown (Sire et al. 2012). Despite the heterogeneity of amelogenin transcripts, there is typically a predominant mRNA transcript that encodes the “major” amelogenin‐secreted protein (isoform), which has about 180 amino acids, is about 25% proline and 15% glutamine in amino acid composition with a single phosphoserine (usually Ser^16^), no glycosylations, and may be divided into three folding units (Goto et al. 1993). Amelogenins have conserved N‐ and C‐terminal sequences, but the middle segment of the protein is often expanded by repetitive sequences that do not seem to interfere with amelogenin function. Examples include the bovine (197 amino acids) (Shimokawa et al. 1987) and opossum (202 amino acids) (Ryu et al. 1998) major amelogenin proteins. The most consistently observed alternatively spliced amelogenin transcript encodes LRAP (Leucine Rich Amelogenin Protein), which is expressed at low levels relative to the major amelogenin and essentially deletes the middle segment (Yuan et al. 1996).

When the mouse amelogenin gene was replaced with the cDNA encoding only the major amelogenin (which could not undergo alternative splicing to generate any of the other amelogenin isoforms), there were no discernable alterations in enamel architecture, incisor morphology, or in the capacity to masticate food (Snead et al. 2011). A statistically significant increase in enamel hardness and decrease in toughness were detected, but these differences did not alter the functionality of the enamel in any detectable way. Amelogenins translated from alternatively spliced transcripts may simply do no harm when expressed at low levels. Overexpression of alternatively spliced amelogenin transcripts can be harmful. A splice junction mutation that increased the inclusion of the normally skipped Exon 4 resulted in X‐linked amelogenesis imperfecta (Cho et al. 2014). There have been many reports claiming the importance of selected amelogenin alternative splicing products, but the production of fully functional enamel in knock‐in mice that only express the major amelogenin isoform argues otherwise.

Amelogenin is partially degraded following its secretion (Fincham et al. 1991). Amelogenin cleavage products accumulate in secretory‐stage enamel, and are slowly reabsorbed into ameloblasts. Matrix metalloproteinase 20 (MMP20) (Bartlett et al. 1996) is a tooth‐specific protease that is secreted concurrently with amelogenin and cleaves amelogenin in vitro at the same sites that amelogenin is known to be cleaved in vivo (Ryu et al. 1999). In *Mmp20* ^*−*/*−*^ mice, which exhibit severe enamel defects, only intact amelogenins and ameloblastin are observed in the secretory‐stage enamel (Yamakoshi et al. 2011).

The major mouse amelogenin (M180) interacts with LAMP1, whereas CD63 interacts with multiple amelogenins, ameloblastin, and enamelin (Zou et al. 2007). The amelogenin receptors LAMP1 and CD63 are membrane markers for late endosomes and lysosomes, and participate in endocytosis (Shapiro et al. 2007), which is a vital process that is necessary for the resorption of all secreted enamel proteins. Blocking LAMP3 decreased amelogenin uptake, suggesting it too facilitates amelogenin reabsorption and degradation (Xu et al. 2008).

Previous studies of amelogenin null mice were conducted on mice maintained in a mixed genetic background (C57BL/6 × 129/SvJ) (Gibson et al. 2001; Hatakeyama et al. 2003); however, genetic background has since been shown to have a significant influence on dental phenotype (Li et al. 2013). Average enamel thickness and enamel mineral density both varied significantly among wild‐type and *Amelx* ^*−*/*−*^ mice of different genetic backgrounds. In this study we bred the *Amelx* null gene into the C57BL/6 background and expanded the characterization of the *Amelx* ^*−*/*−*^ and *Amelx* ^+/*−*^ mice to improve our understanding of the roles amelogenin plays during amelogenesis. In the amelogenin knockout mouse, only Exon 2 was replaced (Gibson et al. 2001). This exon encodes the transcription initiation codon, the signal peptide, the signal peptide cleavage site, and the two N‐terminal amino acids of the secreted protein. Exon 2 is found on all amelogenin mRNA transcripts, is not normally skipped by alternative splicing, and is critical for amelogenin expression and secretion. In the *Amelx* knockout mouse the altered Exon 2 is frequently deleted during splicing, but the resulting transcripts do not generate an amelogenin protein product, so the *Amelx* ^*−*/*−*^ mice are functionally amelogenin null mice (Gibson et al. 2001).

## Materials and Methods

### Ethical compliance

All procedures involving animals were reviewed and approved by the IACUC committee at the University of Michigan (UCUCA).

### Breeding the *Amelx* knockout gene into the C57BL/6 background

*Amelx* null mice in the C57BL/6‐129/SvJ mixed‐genetic background (Gibson et al. 2001) were mated with C57BL/6 mice for at least seven generations to obtain *Amelx* ^*−/−*^, *Amelx* ^*+/−*^, and *Amelx* ^*+/+*^ mice in the C57BL/6 background. Genotyping was done using primers annealing to Exon 2 (CATGGGGACCTGGATTTTGTTTG) and Exon 6 (TCCCGCTTGGTCTTGTCTGTCGCT).

### Dissecting microscopy

Seven‐week‐old mice were anesthetized with isoflurane, sacrificed, and perfused with 4% paraformaldehyde (PFA) for 10 m. Their mandibles were denuded of soft tissues, post‐fixed by immersion in 4% PFA overnight, and rinsed with phosphate‐buffered saline (PBS) three times, for 5 min each. The teeth were cleaned with 1% bleach (sodium hypochlorite), rinsed with PBS, air dried, displayed on the Nikon SMZ1000 dissection microscope, and photographed using a Nikon DXM1200 digital camera.

### Protein extraction and analyses from mouse molars

The protocol used for the mouse molars protein extraction and analysis was previously described (Yamakoshi et al. 2011). Postnatal day 5 (D5) first molars were extracted and separated into soft tissue containing the enamel organ epithelia (EOE) and the dental hard tissue. The hard tissue from the four molars collected from each mouse was incubated in 1 mL of 0.17 N HCl/0.95% formic acid for 2 h at 4°C. Undissolved material was removed by centrifugation. The supernatant containing the crude protein extract in strong acid buffer was exchanged with 0.01% formic acid using a centrifugal 3K‐filter unit (UFC800324; Amicon by EMD Millipore, Billerica, MA). The EOE was incubated in NP40 Cell Lysis Buffer (Thermo Fisher Scientific, Waltham, MA) with protease inhibitors, sonicated, and incubated in 0.5% formic acid overnight. These samples were used for sodium dodecyl sulfate polyacrylamide gel electrophoresis (SDS‐PAGE), Coomassie Brilliant Blue (CBB) staining, and amelogenin immunoblotting. The amount of protein applied per 1X lane for SDS‐PAGE was 1/6 of a tooth. Polyclonal rabbit anti‐full‐length mouse recombinant amelogenin antibody (rM179; 1:2000) was used for amelogenin immunostaining, and ECL prime Western blotting detection reagent (RPN2232; GE Healthcare Life Sciences, Piscataway, NJ) was used for visualization.

### RT‐PCR analysis of amelogenin expression

The first molars of 6‐day‐old *Amelx* ^*+/+*^ and *Amelx* ^*−/−*^ mice were extracted, manually homogenized, and RNA isolated using the Dynabeads mRNA DIRECT™ Micro Kit (Thermo Fisher Scientific). First‐strand cDNA was synthesized at 42°C for 25 min using an oligo(dT)_16_ primer. PCR amplification was performed using primers for Exon 2 (AATGGGGACCTGGATTTTGTTTG) and Exon 6 (TCCCGCTTGGTCTTGTCTGTCGCT) using the GeneAmp RNA PCR core kit (PE Biosystems, Foster, CA).

### Backscattered scanning electron microscopy

The backscattered scanning electron microscopy (bSEM) procedures were described previously (Smith et al. 2011b). Left and right hemi‐mandibles of 7‐week‐old *Amelx* ^*+/+*^, *Amelx* ^*+/−*^, and *Amelx* ^*−/−*^ mice were dissected free of soft tissue. The hemi‐mandibles were dehydrated with an acetone series (30, 50, 70, 80, 90, and 100%), embedded in epoxy, cross sectioned at 1 mm increments along their lengths, and characterized by bSEM at each level. Level 8 cross sections, which are even with the buccal crest of alveolar bone, were used to measure enamel thickness and nanohardness.

Whole surface incisor imaging was done at 7 weeks and whole surface molar imaging was performed at D14 on *Amelx* ^*+/+*^, *Amelx* ^*+/−*^, and *Amelx* ^*−/−*^ mice. For incisor imaging, the soft tissue and bony caps covering the mandibular incisors were removed, and the incisors were examined at 50× magnification using a Hitachi S‐3000N variable pressure scanning electron microscope in the backscatter mode at 25 kV and 20 pascal pressure.

For molar imaging, *Amelx* ^*+/+*^, *Amelx* ^*+/−*^, and *Amelx* ^*−/−*^ mouse molars were prepared as follows: D14 mandibles were submerged in 4% PFA overnight, and the following day, hemi‐mandibles were carefully dissected of soft tissues, submerged in 1% NaClO for 20 min, rinsed, and dehydrated using an acetone series (as described above). The hemi‐mandibles were mounted on metallic stubs using conductive carbon cement, then examined using a Hitachi S‐3000N variable pressure scanning electron microscope (Century City, Los Angeles, CA) in the backscatter mode.

### Scanning electron microscopy

Scanning electron microscopy (SEM) evaluation was performed at the University of Michigan Microscopy and Image Analysis Laboratory (Ann Arbor, MI). Acetone‐dehydrated, air‐dried hemi‐mandibles and mandibular incisors from 7‐week‐old *Amelx* ^*+/+*^, *Amelx* ^*+/−*^, and *Amelx* ^*−/−*^ mice were fractured at Level 8, mounted on metallic stubs using conductive carbon cement, and sputter coated with an Au‐Pd film to increase conductivity. An Amray EF 1910 Scanning Electron Microscope operating at an accelerating voltage of 5 kV was used to image the samples.

### Nanohardness testing

Hemi‐mandibles from Amelx^+/+^, Amelx^+/*−*^, and Amelx^*−*/*−*^ mice were collected at 7 weeks. Left and right hemi‐mandibles *Amelx* ^*+/+*^, *Amelx* ^*+/−*^, and *Amelx* ^*−/−*^ mice were dissected free of soft tissue, the hemi‐mandibles were dehydrated with an acetone series (30, 50, 70, 80, 90, and 100%), and embedded in epoxy. The embedded hemi‐mandibles/incisors were cut transversely at the level of the labial alveolar crest (Level 8) and reembedded in Castolite AC in 25‐mm SeriForm molds (Struers Inc., Westlake, OH). The incisor cross sections were successively polished with 400, 800, and 1200 grit waterproof silicon carbide papers, followed by polishing with 1‐micron diamond paste. Nanohardness testing was done using a Hysitron 950 Triboindenter with a nanoDMA transducer and Berkovich probe, and the nanoindentations were analyzed using Triboscan 9 software (University of Michigan Center for Materials Characterization).

### Transmission Electron Microscopy

Seven‐week‐old *Amelx* ^*+/+*^, *Amelx* ^*−/−*^, and *Enam* ^*−/−*^ mice were deeply anesthetized using isoflurane and perfused with 5% glutaraldehyde in sodium cacodylate buffer for 20 min. Mandibles were dissected, cleansed of soft tissue, post‐fixed with 1% reduced osmium tetroxide for 2 h, and dehydrated using an acetone gradient. Mandibular incisors were sectioned into 70‐nm sections and floated on oil using an ultrathin microtome. The sections were stained with uranyl acetate, then lead citrate, and viewed by transmission electron microscopy (TEM) using a Philips CM‐100 transmission electron microscope (University of Michigan Microscopy & Image Analysis Laboratory).

### Incisor histology

*Amelx* ^*+/+*^, *Amelx* ^*+/−*^, and *Amelx* ^*−/−*^ mice at 7 weeks were deeply anesthetized with isoflurane, fixed by cardiac perfusion with 5% glutaraldehyde in 0.1 mol/L sodium cacodylate buffer (pH 7.2–7.4) containing 0.05% calcium chloride, postfixed for 2 h at 4°C, and rinsed 3× for 15 min each with 0.1 mol/L sodium cacodylate buffer. The samples were decalcified at 4°C by immersion in 1 L of 4.13% disodium ethylenediaminetetraacetic acid (EDTA, pH 7.3), with agitation, and the EDTA solution was changed every other day for 30 days. The samples were then washed in PBS at 4°C 4–5 times every 0.5–1 h, washed overnight, postfixed for 2 h in 1% osmium tetroxide in 1.5% potassium ferrocyanide, and dehydrated using an acetone gradient. The samples were then embedded in Epon812 substitute and semithin sectioned and stained with 0.1% toluidine blue as described elsewhere (Smith et al. 2011a). At least three mandibular incisors were processed for longitudinal sectioning, and three mandibular incisors were processed for cross sectioning at 1 mm increments for amelogenin immunohistochemistry.

### Amelogenin immunohistochemistry

Seven‐week mandibular incisor cross sections were collected as described above underwent regular immunostaining and image processing as previously described (Wang et al. 2014). The primary antibody was polyclonal rabbit anti‐full‐length mouse recombinant amelogenin antibody (rM179; 1:2000), and the secondary antibody was anti‐rabbit IgG conjugated with Alexa Fluor 488 (1:500, A11034; Invitrogen, Carlsbad, CA).

### Molar histology

This histology protocol was previously described (Wang et al. 2015). Day 5 and 11 mouse heads were quickly dissected free of skin, cut in half, and immersed in 4% PFA fixative overnight at 4°C, washed in PBS 4–5 times (every 0.5–1 h) at 4°C, and decalcified at 4°C by immersion in 1 L of 4.13% disodium EDTA (pH 7.3) with agitation. The EDTA solution was changed every other day for 8–9 days for D5 mice and 19–21 days for D11 mice. The samples were washed in PBS at 4°C, 4–5 times (every 0.5–1 h) followed by one overnight wash. The samples were dehydrated using a graded ethanol series followed by xylene, embedded in paraffin, sectioned at 5‐*μ*m thickness, spread on a water bath (52°C), loaded on plus gold glass slides (Thermo Fisher Scientific), dried at room temperature overnight, and stained with hematoxylin and eosin (H&E) stain.

### X‐ray diffraction

Incisors from 7‐week‐old *Amelx* ^+/+^ and *Amelx* ^*−*/*−*^ mice were dissected free of the mandibles immediately after sacraficing and freeze dried using liquid nitrogen, embedded in Castolite AC (Eager Polymers, Chicago, Il), and cross sectioned at Level 6, which is ~6 mm from the basal end of the incisor, and 1 mm basally and mounted for X‐ray analyses. X‐ray diffraction measurements were performed at the Advanced Photon Source (APS) at the Argonne National Laboratory, using the hard X‐ray microdiffraction facility at Beamline 2‐ID‐D (Cai et al. 2000). The X‐ray radiation used in this study was generated from a 7 GeV electron beam and an APS undulator A (Dejus et al. 2002) in the storage ring. X‐rays with energy of 10.1 keV (wavelength = 0.1228 nm) were selected by a double‐crystal Si <111> monochromator. Through a gold zone‐plate focusing optics, the X‐ray beam was focused into a spot of size 200 nm and delivered to the sample with a flux ~3 × 10^9^ photons s^−1^ (Cai et al. 2000). The sample was mounted on and its angular position was manipulated by a six‐circle kappa geometry diffractometer (Libera et al. 2002). A Rayonix Mar165 CCD detector, with 2048 × 2048 pixels and 80‐micron pixel size, was mounted about 80 mm downstream of the sample to collect diffraction signals.

## Results

**Figure 1:**
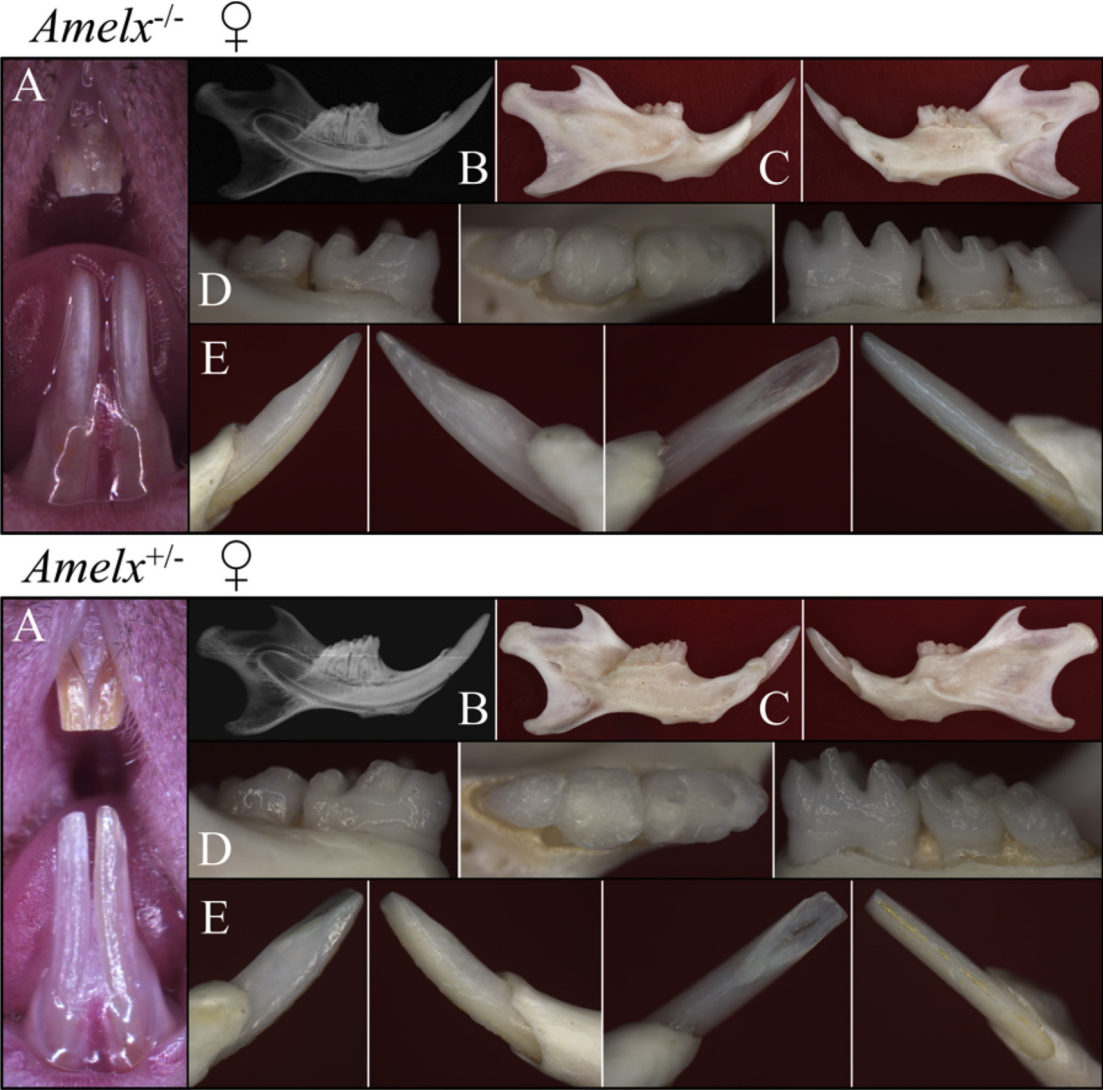
Oral photos of 7‐week null (*Amelx* ^*−*/*−*^) and heterozygous (*Amelx* ^+/*−*^) mice. (A) Frontal view of incisors *in situ*. (B) Radiograph of right hemi‐mandible. (C) Right and left hemi‐mandibles following removal of soft tissues. (D) Buccal, occlusal, and lingual views of mandibular molars. (E) Lateral, mesial, lingual, and facial views of a mandibular incisor. The *Amelx* ^*−*/*−*^ enamel is thin and relatively smooth, but undergoes rapid attrition on working surfaces. The *Amelx* ^+/*−*^ enamel shows ridges of thick and thin enamel, giving the enamel a wrinkled appearance. Oral photos of the *Amelx* ^+/+^ enamel are shown in the Appendix S2.

The C57BL/6‐129/SvJ *Amelx* null mice were crossed with C57BL/6 mice for a minimum of seven generations. The mice of all three genotypes (*Amelx* ^*−*/*−*^, *Amelx* ^+/*−*^, and *Amelx* ^+/+^) were healthy, viable, and fertile. For the initial assessment, *Amelx* ^*−*/*−*^, *Amelx* ^+/*−*^, and *Amelx* ^+/+^ incisors at 7 weeks were photographed, the mandibles were stripped of soft tissue, photographed and radiographed, and the incisors and molars were photographed under a dissecting microscope (Fig. 1; Appendix S2). The *Amelx* ^*−*/*−*^ enamel was thin and smooth, and had undergone significant posteruption attrition on the labial surface of the mandibular incisors and on the molar cusp tips (Fig. 1, top). The *Amelx* ^*−*/*−*^ and *Amelx* ^+/+^ mice could be easily distinguished from each other simply by inspecting their enamel. The *Amelx* ^+/*−*^ genotype is only possible in females, as the amelogenin gene localizes to the X chromosome (so males have only one copy of the amelogenin gene in mice). The *Amelx* ^+/*−*^ enamel was visibly thicker than the *Amelx* ^*−*/*−*^ enamel, and did not undergo significant attrition, but their molars and incisors exhibited a characteristic vertical banding pattern caused by alternating bands of thick and thin enamel (Fig. 1, bottom). The *Amelx* ^+/*−*^ genotype in most females could be accurately inferred by a vertical banding pattern on the enamel; however, the *Amelx* ^+/*−*^ enamel on any given incisor could exhibit the full spectrum of enamel phenotypes: from thin (like *Amelx* ^*−*/*−*^ enamel), banded, or apparently normal (like *Amelx* ^+/+^ enamel). There was no apparent recession of alveolar bone or loss of periodontal attachment, and radiographs revealed normal root form for the *Amelx* ^*−*/*−*^ and *Amelx* ^+/*−*^ mice relative to the wild‐type (*Amelx* ^+/+^) mice.

Western blot analyses were performed on proteins extracted from D5 first molars, which confirmed that no amelogenin protein was synthesized in *Amelx* ^*−*/*−*^ mice (Appendix S3A) (Gibson et al. 2001). RT‐PCR of RNA isolated from tissue surrounding the first molar roots of 6‐month‐old mice detected no amelogenin transcripts, whereas the control using RNA isolated from D5 first molar enamel organ epithelia (EOE) was positive (Appendix S3B). This confirms previous results showing no amelogenin expression during tooth root development (Luo et al. 1991; Hu et al. 2001; Nishio et al. 2010).

### Mandibular incisors

**Figure 2:**
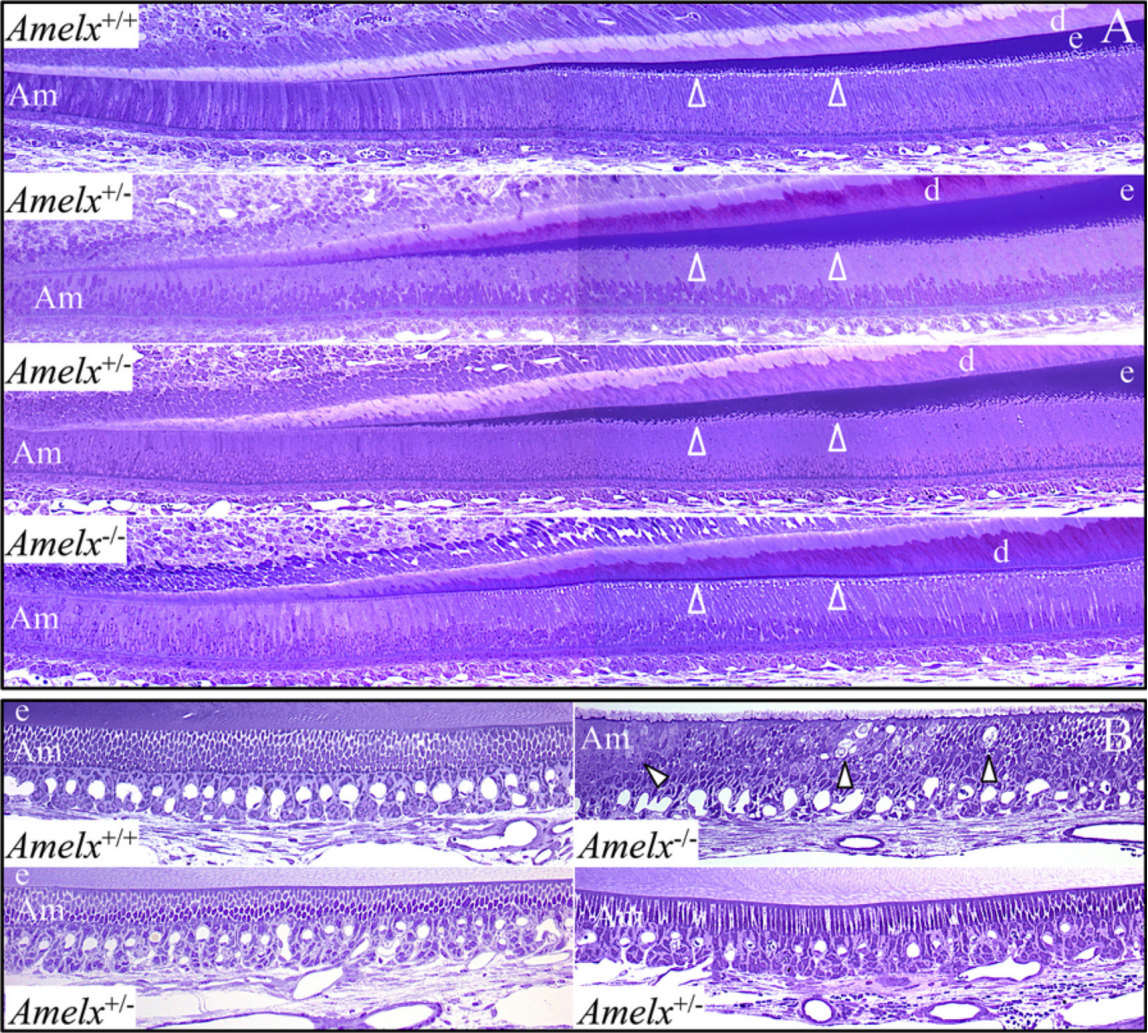
Longitudenal mandibular incisor histology at 7 weeks. (A) Sections showing the onset of mineralization, and initial and secretory‐stage enamel formation. *Amelx* ^+/+^ enamel steadily increases in thickness. *Amelx* ^+/*−*^ enamel increases in thickness are more stepped. *Amelx* ^*−*/*−*^ enamel appears to initiate normally but remains thin. The ameloblasts look similar in all genotypes, except that Tomes’ processes penetrating into the more deeply stained enamel layer (arrowheads) are not evident. Key: Am, ameloblasts; d, dentin; e, enamel. (B) Sections showing maturation‐stage ameloblasts. Pathology is evident in the layer of maturation‐stage ameloblasts (arrowheads). The complete longitudinal histological surveys of *Amelx* ^*−*/*−*^ and *Amelx* ^+/*−*^ are provided in Appendices S4–S8.

**Figure 3:**
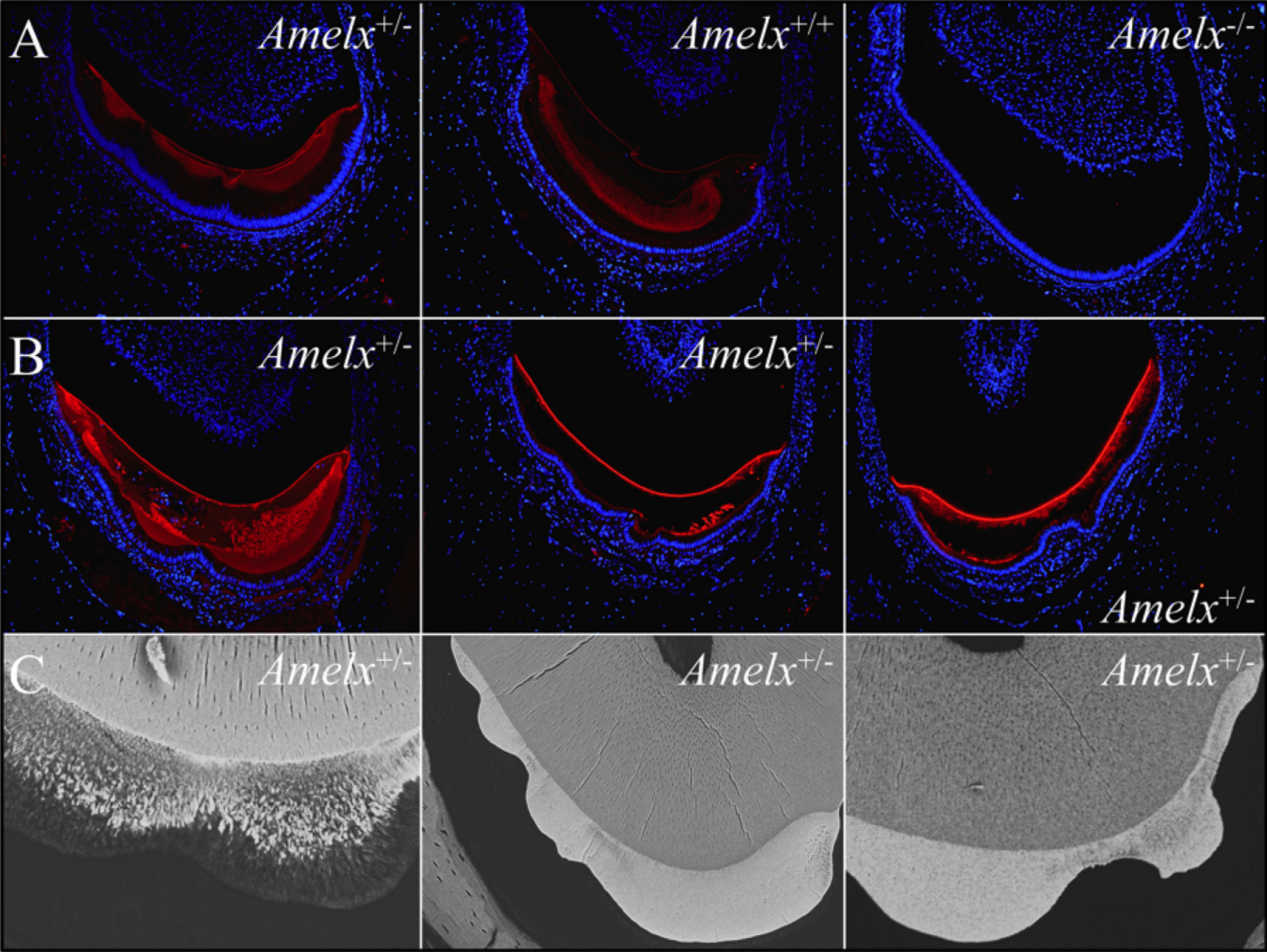
Amelogenin Immunohistochemistry of DAPI‐stained 7‐week mandibular incisor cross sections. Row A: Female heterozygous (*Amelx* ^+/*−*^) at level 1; Wild type (*Amelx* ^+/+^) and Null (*Amelx* ^*−*/*−*^) at level 3. Row B: Female heterozygous (*Amelx* ^+/*−*^) at levels 3, 5, and 7. Positive signal for amelogenin (red) is thicker where the enamel layer is thicker. Row C: Female heterozygous (*Amelx* ^+/*−*^) from levels 3, 8, and 8 that show a similar lyonization pattern as the IHC samples.

Longitudinal sections of mandibular incisors were characterized by light microscopy (Fig. 2 and Appendices S4–S8). Secretory‐stage ameloblasts were similar in *Amelx* ^+/+^, *Amelx* ^+/*−*^, and *Amelx* ^*−*/*−*^ mandibular incisors and did not suffer the kinds of major pathological changes that were evident in the *Enam* and *Ambn* knockout mice (Fukumoto et al. 2004; Hu et al. 2011); however, Tomes’ processes were not observed penetrating into the surface of the thin *Amelx* ^*−*/*−*^ enamel. Immunohistochemistry of *Amelx* ^+/+^, *Amelx* ^+/*−*^, and *Amelx* ^*−*/*−*^ mandibular incisor cross sections using affinity‐purified polyclonal antibodies raised against recombinant mouse amelogenin (Simmer et al. 1994b) confirmed the absence of amelogenin protein in *Amelx* ^*−*/*−*^ enamel (Fig. 3). Amelogenin levels varied in the *Amelx* ^+/*−*^ incisors, with more amelogenin apparent where the enamel had grown thicker, and almost no amelogenin where the enamel was very thin. These observations support the hypothesis that variations in the thickness of enamel in heterozygous females with X‐linked amelogenesis imperfecta are the result of lyonization.

**Figure 4:**
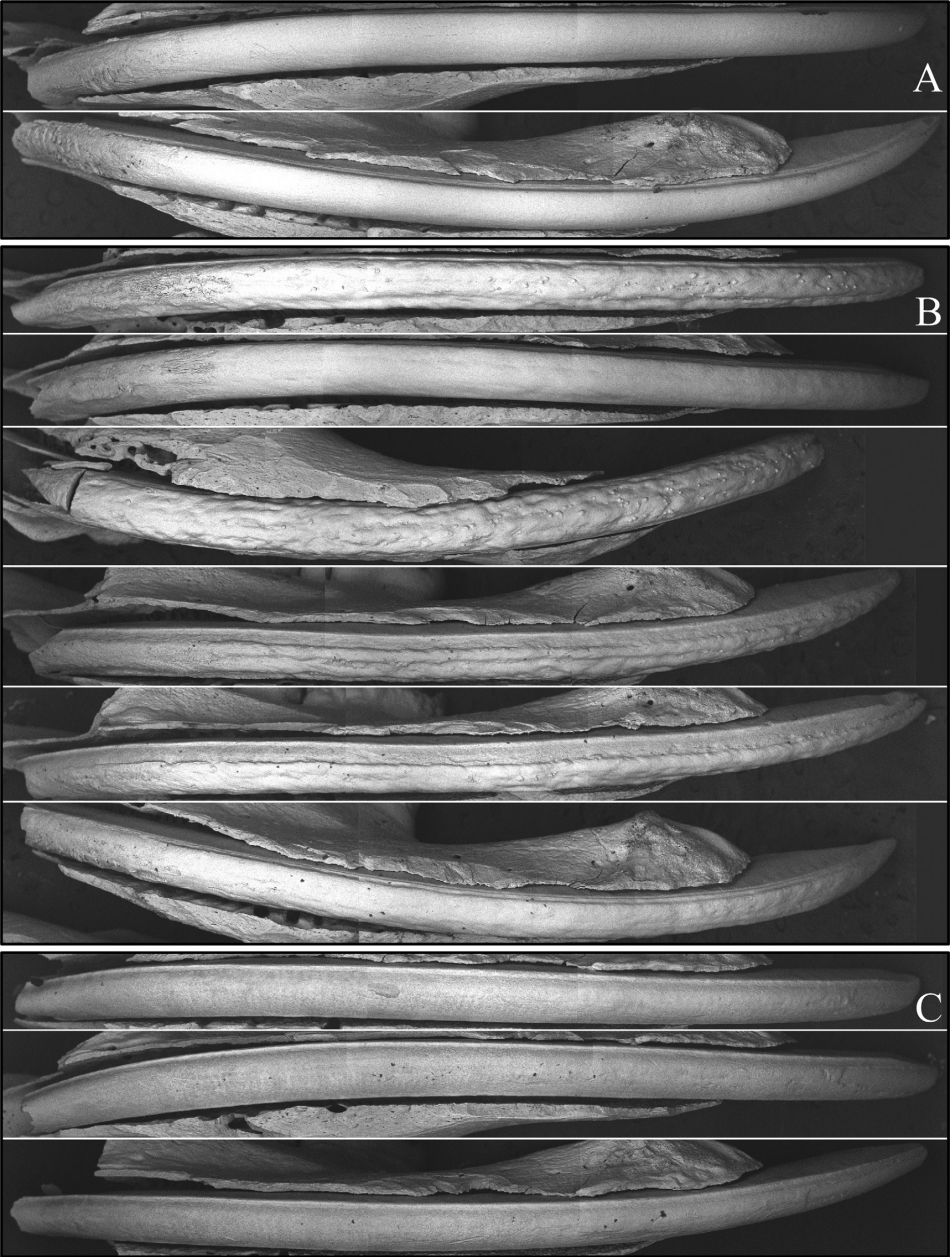
Backscatter electron microscopy of 7‐week mandibular incisor facial surfaces. (A) Wild type (*Amelx* ^+/+^). (B) Female heterozygous (*Amelx* ^+/*−*^). (C) Null (*Amelx* ^*−*/*−*^). The *Amelx* ^*−*/*−*^ incisor enamel is relatively smooth, with few apparent surface nodules. The *Amelx* ^+/*−*^ incisor enamel is highly irregular areas of hypoplastic enamel sometimes spanning the entire length of the incisor. The basal end (secretory stage) is on the left. The incisal end is on the right.

The enamel surfaces of mandibular incisors were examined by backscattered electron microscopy (Fig. 4). The *Amelx* ^+/+^ enamel surface was smooth and even, except basally in the region corresponding to early enamel formation. The *Amelx* ^+/*−*^ enamel surface was typically rough with longitudinal grooves of thin enamel alternating with ridges of thicker enamel. These grooves extended all the way from the basal to incisal ends of the mandibular incisor, indicating that they originally formed during the secretory stage. Surface nodules, or mineralized bumps on the enamel surface, were often arrayed longitudinally along the maturation‐stage enamel surface, particularly near a junction where a depression met a ridge. The nodules were only rarely observed in secretory‐stage enamel. Attrition of the enamel at the incisal edge was always observed in *Amelx* ^*−*/*−*^ mandibular incisors. Inspection of *Amelx* ^+/+^, *Amelx* ^+/*−*^, and *Amelx* ^*−*/*−*^ mandibular molar roots by SEM did not detect any significant root resorption, although root resorption was evident in the original F3 (mixed background) molars, including the wild‐type mice (Appendix S9). This finding of no defects on the roots of C57BL/6 *Amelx* ^*−*/*−*^ mice is consistent with previous data showing that amelogenin is not expressed during tooth root formation, that dentin, cementum, and the periodontal ligament are all structurally normal in the *Arhgap6*/*Amelx* double null mice (Prakash et al. 2005), and the lack of amelogenin selection pressure in enamel‐less mammals that still make teeth attached by a periodontal ligament (Meredith et al. 2014).

**Figure 5:**
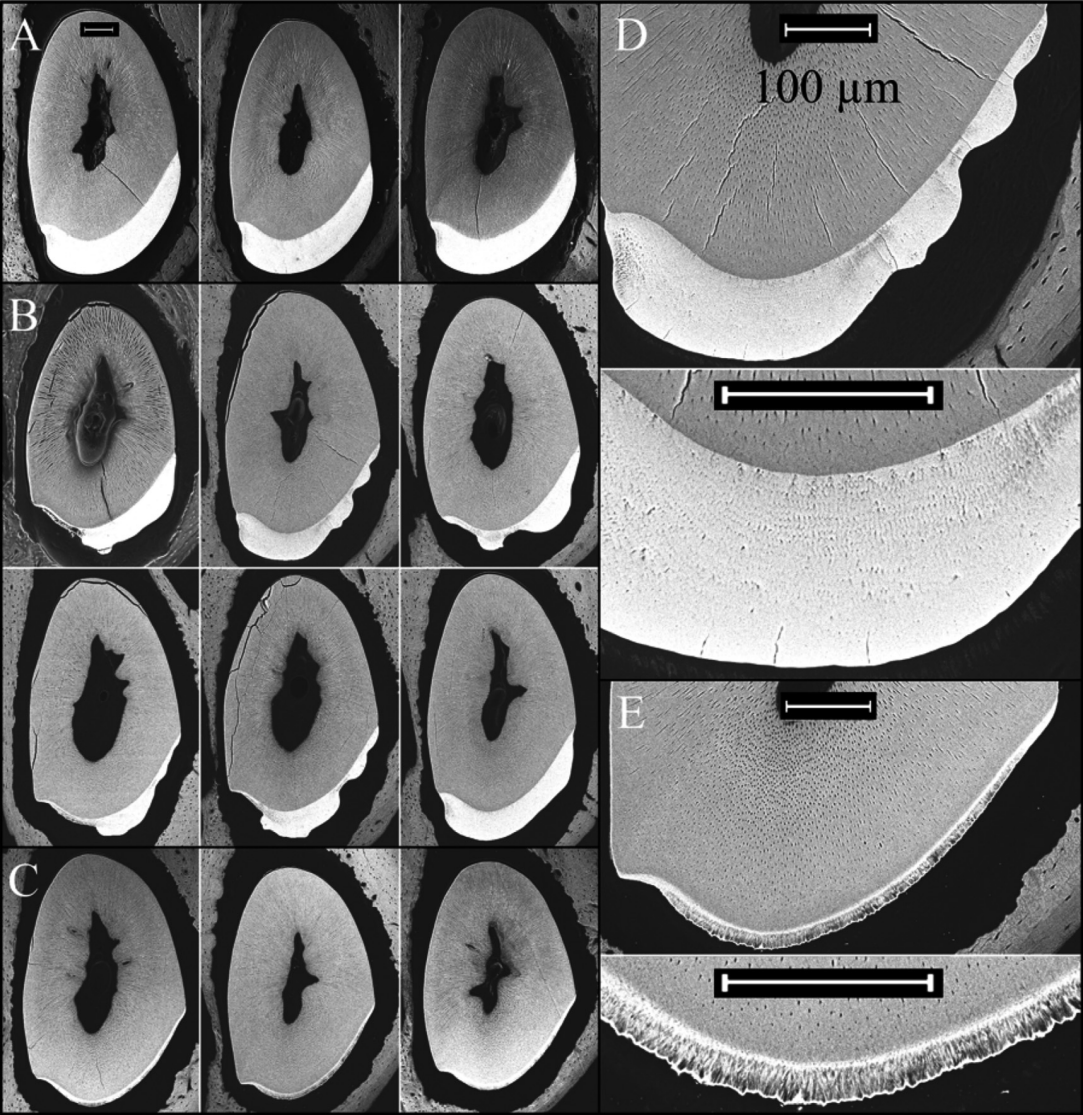
Backscatter electron microscopy of mandibular incisor Level 8 cross sections (at the level of the buccal alveolar crest). (A) Three wild type (*Amelx* ^+/+^). (B) Six heterozygous (*Amelx* ^+/*−*^) female mice. (C) Three null (*Amelx* ^*−*/*−*^) mice. (D) Higher‐magnification views of female heterozygous (*Amelx* ^+/*−*^) enamel. (E) Higher‐magnification views of null (*Amelx* ^*−*/*−*^) enamel. Scale bars = 100 *μ*M. Note that the enamel in the female heterozygous (*Amelx* ^+/*−*^) mice varies greatly in thickness, ranging from null to wild‐type levels. bSEM images of incisor cross sections from four *Amelx* ^*−*/*−*^ mice (Appendices S10–S13); five *Amelx* ^+/*−*^ mice (Appendices S14–S18), and one wild‐type (*Amelx* ^+/+^) mouse (Appendix S19).

*Amelx* ^*−*/*−*^, *Amelx* ^+/*−*^, and *Amelx* ^+/+^ incisors at 7 weeks were cross sectioned at 1‐mm increments along their lengths and each level was characterized by bSEM (Appendices S10–S19). Level 8 sections from this series are shown in Figure 5. Level 8 was chosen for display because this section is even with the labial crest of alveolar bone, where enamel maturation is advanced, but where this portion of the incisor has not yet erupted into the oral cavity where it might be altered. The *Amelx* ^*−*/*−*^ enamel layer was uniformly thin and most highly mineralized near the dentino‐enamel junction (DEJ). The enamel away from the DEJ varied in density but was clearly less dense than normal enamel. The *Amelx* ^*−*/*−*^ enamel layer was not homogeneous in density, but appeared to have a repeating substructure, so that the cross‐sectioned enamel layer resembled a picket fence. This pattern gave the impression that the enamel was comprised of short enamel rods, but this was only an impression, as decussating rods like those found in wild‐type enamel were not observed in *Amelx* ^*−*/*−*^ enamel. The *Amelx* ^+/*−*^ enamel was characterized by random variations in thickness, ranging from being as thin as *Amelx* ^*−*/*−*^ enamel to as thick as *Amelx* ^+/+^ enamel in different locations on a single tooth. Where the enamel was thick, it reached high density like normal enamel, and exhibited decussating rod structures. In early maturation‐stage bSEM sections of *Amelx* ^+/*−*^ incisors (Appendices S14–S18), before the enamel had matured and reached a more uniform density, the enamel often showed two distinct layers. In some cases the differences between these two layers appeared to reflect the normal distinction between inner/middle enamel and the outer enamel. In other cases the most superficial mineralized layer looked more pathological, like that of surface nodules.

*Amelx* ^*−*/*−*^ and *Amelx* ^+/+^ Level 8 cross sections were used to measure enamel thickness and nanohardness. Enamel thickness was measured at the height of contour of 6 *Amelx* ^*−*/*−*^ and 6 *Amelx* ^+/+^ incisors (Appendix S20). The wild‐type enamel averaged 122.3 ± 7.9 *μ*m in thickness; the null enamel averaged 20.3 ± 3.3 *μ*m. Thus, the null enamel was only 1/6^th^ as thick as the wild‐type enamel. The *Amelx* ^+/*−*^ enamel thickness was too variable to measure.

**Figure 6:**
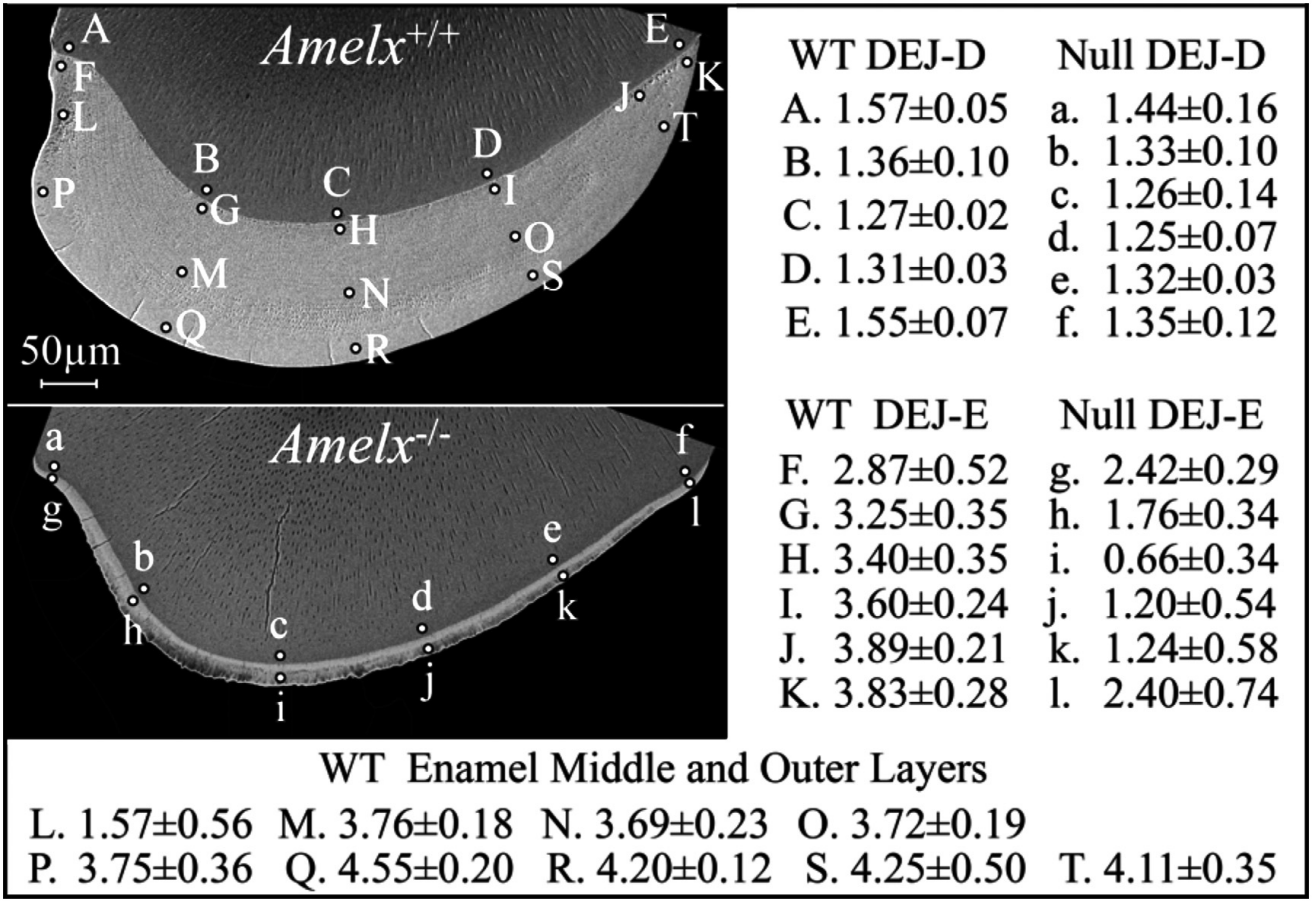
*Amelx* ^+/+^ and *Amelx* ^*−*/*−*^ nanohardness testing. Backscatter electron microscopy images of *Amelx* ^+/+^ and *Amelx* ^*−*/*−*^ mandibular incisor cross sections from the level of the labial alveolar crest showing the sections tested and the target sites (labeled dots) for nanoindenting. The average and standard deviations of the hardness measurements for each indent site from six independent samples are shown (in gigapascal; Gpa). The average hardness value for the combined enamel indents was 3.63 ± 0.75 Gpa for *Amelx* ^+/+^ and 1.61 ± 0.80 Gpa for *Amelx* ^*−*/*−*^. The *Amelx* ^*−*/*−*^ enamel hardness near the cervical margins (2.41 ± 0.54 Gpa; points g and l) was nearly twice that of the *Amelx* ^*−*/*−*^ enamel away from the cervical margins (1.22 ± 0.59 Gpa; points h, i, j, and k). The average hardness values for the combined dentin indents were similar in the wild‐type and null incisors: *Amelx* ^+/+^ dentin 1.41 ± 0.14 Gpa; *Amelx* ^*−*/*−*^ dentin 1.32 ± 0.12 Gpa.

Dentin (near the DEJ) and enamel nanohardness measurements were made at various intervals on *Amelx* ^+/+^, *Amelx* ^+/*−*^, and *Amelx* ^*−*/*−*^ Level 8 incisor cross sections (Fig. 6; Appendix S21). Dentin was tested at spaced intervals near the DEJ (Fig. 6A–E for *Amelx* ^+/+^ and a–e for *Amelx* ^+/*−*^). Enamel was also tested at spaced intervals, with separate measurements obtained for the inner, middle, and outer enamel when the enamel layer was sufficiently thick to allow it. The average hardness values for *Amelx* ^+/+^, *Amelx* ^+/*−*^, and *Amelx* ^*−*/*−*^ enamel were 3.63 ± 0.75, 3.46 ± 0.91, and 1.61 ± 0.80 gigapascal (Gpa), respectively. The *Amelx* null enamel was softer away from the cervical margins (1.22 ± 0.59 Gpa). Thus, the overall *Amelx* ^*−*/*−*^ enamel hardness score was only half that of the wild type, while the hardness score of the enamel away from the cervical margins was only 1/3^rd^ that of the wild type. The average hardness values for dentin near the DEJ were similar in the *Amelx* ^+/+^, *Amelx* ^+/*−*^, and *Amelx* ^*−*/*−*^ incisors: 1.41 ± 0.14, 1.39 ± 0.10, and 1.32 ± 0.12 Gpa, respectively, although it trended lower in the absence of amelogenin.

**Figure 7:**
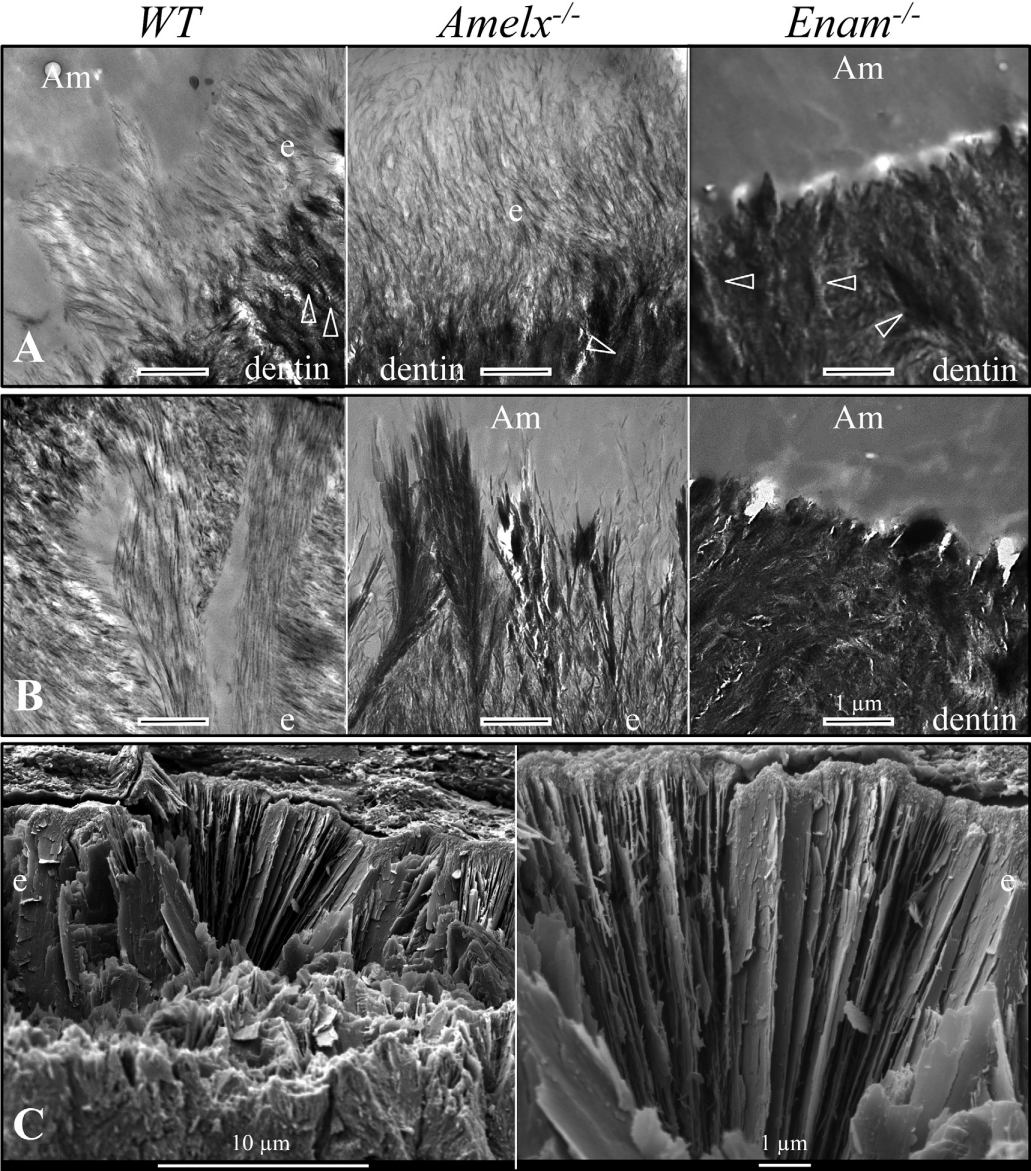
Ultrastructure of enamel mineral ribbons forming in 7‐week mandibular incisors. (A) TEMs showing initial enamel ribbons forming at the DEJ in wild‐type (WT) and *Amelx* ^*−*/*−*^ mice, but not in *Enam* ^*−*/*−*^ mice (which do not make any enamel). The dentin mineral stains darker than the enamel, with banded collagen fibers (arrowheads) oriented toward the ameloblast. (B) TEMs of a more incisal position in the secretory stage. The wild‐type enamel is thicker and organized into rod and interrod structures. The *Amelx* ^*−*/*−*^ enamel ribbons appear to have fused together. No rod–interrod organization was evident. *Amelx* ^*−*/*−*^ enamel ribbons were not separated by weakly staining material. (C) SEMs of *Amelx* ^*−*/*−*^ enamel plates evident after fracturing the incisor at Level 8 (alveolar crest). No decussation pattern was observed. Key: Am, ameloblasts; d, dentin; e, enamel.

The ultrastructure of the early enamel mineral formed in wild‐type, *Amelx* ^*−*/*−*^, and *Enam* ^*−*/*−*^ mice was assessed by transmission electron microscopy of 7‐week mandibular incisors (Fig. 7). The initial enamel formed in wild‐type and *Amelx* ^*−*/*−*^ incisors was comprised of thin ribbons extending from dentin collagen to the ameloblast. In contrast, no enamel ribbons formed in the *Enam* ^*−*/*−*^ mice (Fig. 7A) (Hu et al. 2008, 2014). The enamel ribbons of wild‐type mice became oriented into rod and interrod enamel. In contrast, some enamel ribbons in the *Amelx* ^*−*/*−*^ mice appeared to have fused about 5 *μ*m away from the ameloblast membrane with the unfused tips of the crystals radiating toward the mineralization front like a Japanese fan (Fig. 7B). There was little or no lightly stained material separating the individual crystals in the *Amelx* ^*−*/*−*^ enamel. Sometimes the fans appeared to be oriented in different directions, reminiscent of the decussating pattern of enamel rods. Scanning electron microscopy of a fractured *Amelx* ^*−*/*−*^ mandibular incisor at the level of the alveolar crest (Level 8) showed it to be comprised of long, thin plates radiating toward the enamel surface (Fig. 7C). The radiating system of thin crystal plates may have had their origins in the fan‐like structures that started forming during the secretory stage.

**Figure 8:**
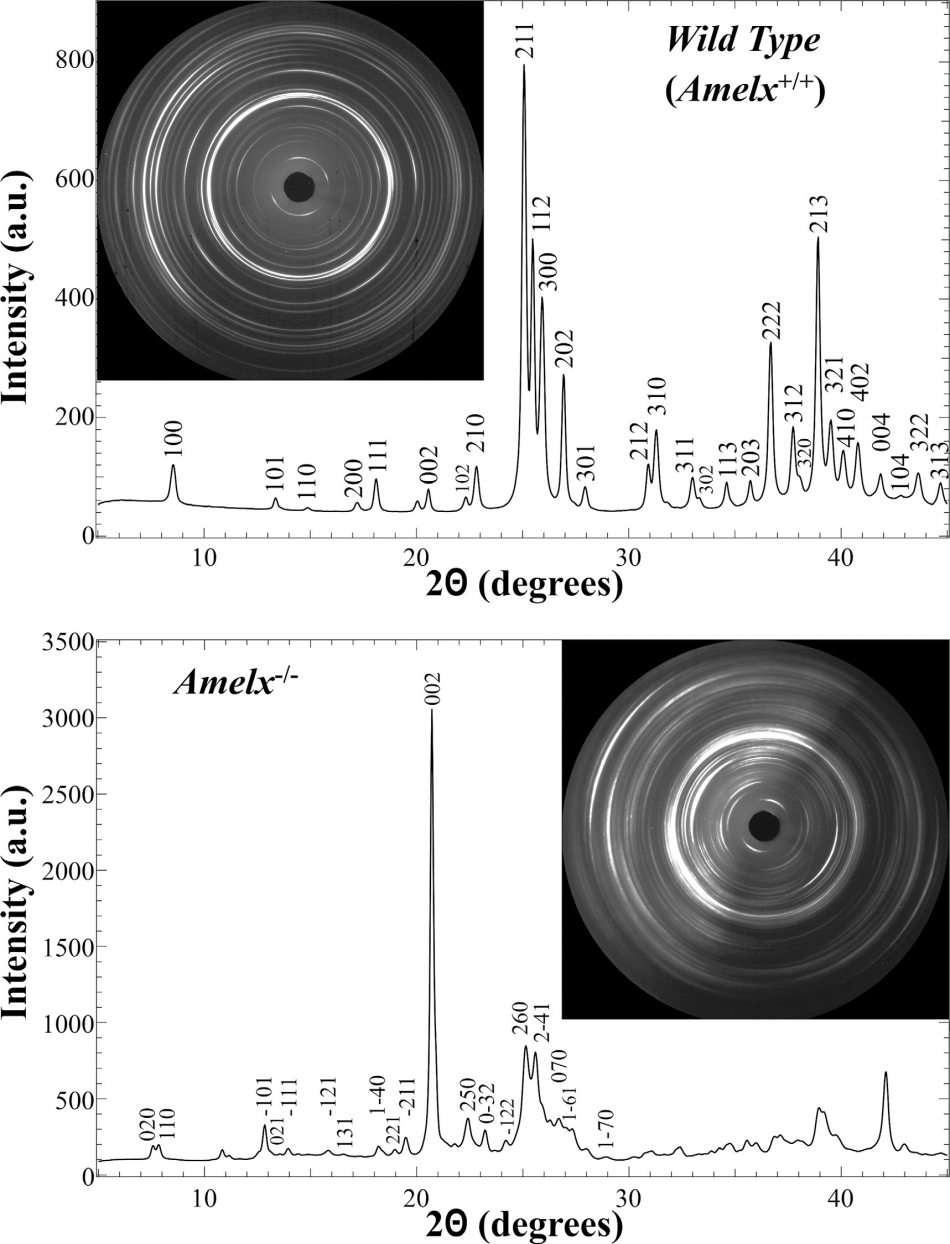
X‐ray (λ=0.1228 nm) diffraction patterns and diffractograms of *Amelx* ^+/+^ and *Amelx* ^*−*/*−*^ mandibular incisor enamel cross sectioned at the level of the labial alveolar crest in 7‐week‐old mice. Top: *Amelx* ^+/+^ diffractogram plotting the intensity of the diffraction (in arbitrary units, a.u.) against the 2theta (2Θ) diffraction angle (in degrees). Above the peaks are Miller indices indicating the crystal plane that produced the diffraction.

The unusual plate‐like pattern of the enamel crystals observed in the *Amelx* ^*−*/*−*^ mandibular incisors prompted us to identify the crystal structure by X‐ray diffraction (Fig. 8). The startling finding was that, unlike wild‐type enamel, which produced a diffraction pattern characteristic of calcium hydroxyapatite (HAP), the *Amelx* ^*−*/*−*^ enamel diffraction pattern matched that of octacalcium phosphate (OCP). This finding was wholly unexpected and forces a thorough reconsideration of amelogenin's role in amelogenesis.

### Mandibular molars

**Figure 9:**
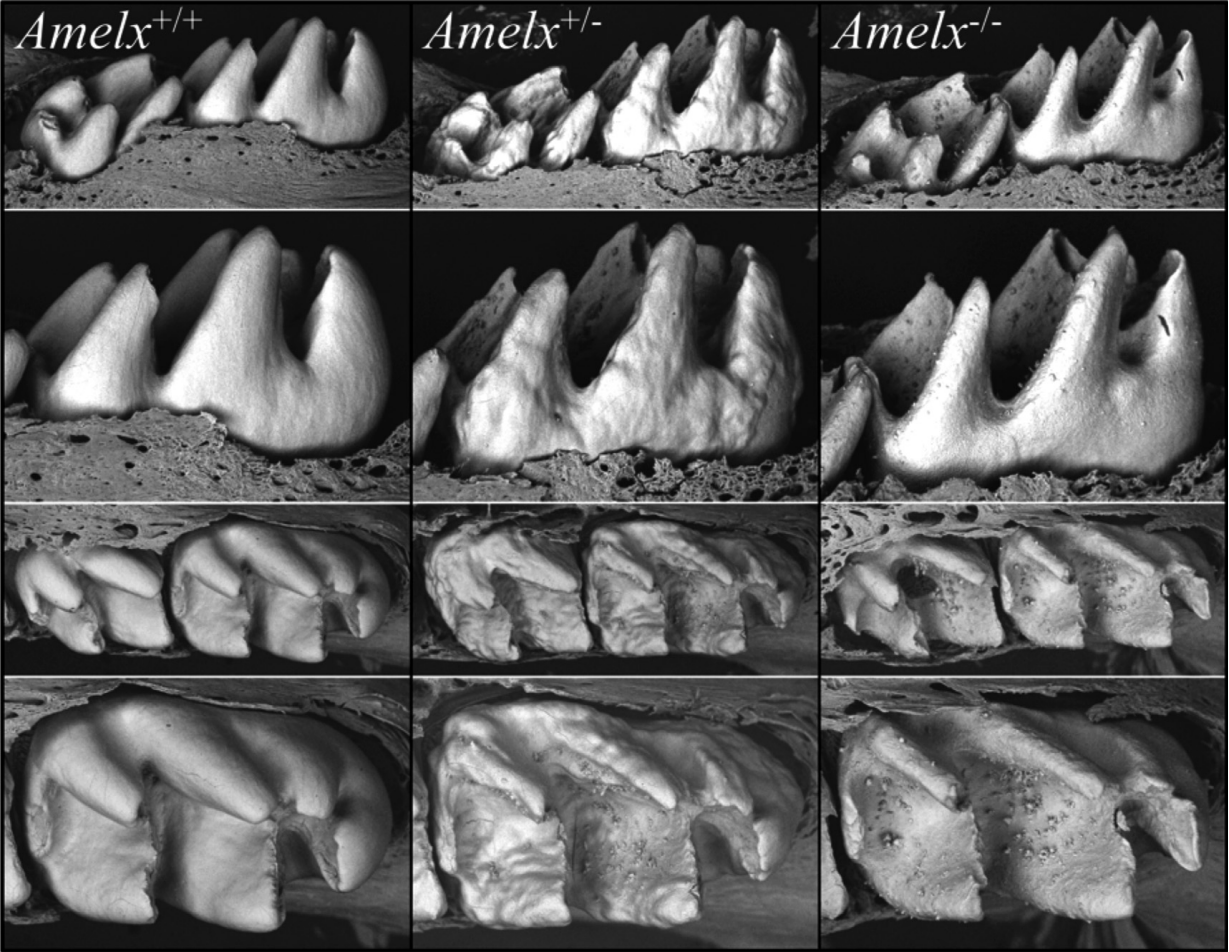
Backscatter electron microscopy of D14 mandibular molars. Wild‐type (*Amelx* ^+/+^) enamel is smooth. Female heterozygous (*Amelx* ^+/*−*^) enamel shows variations in thickness and abundant surface nodules, especially on the occlusal surface. Null (*Amelx* ^*−*/*−*^) enamel is sparse, but the occlusal surface shows abundant nodules.

**Figure 10:**
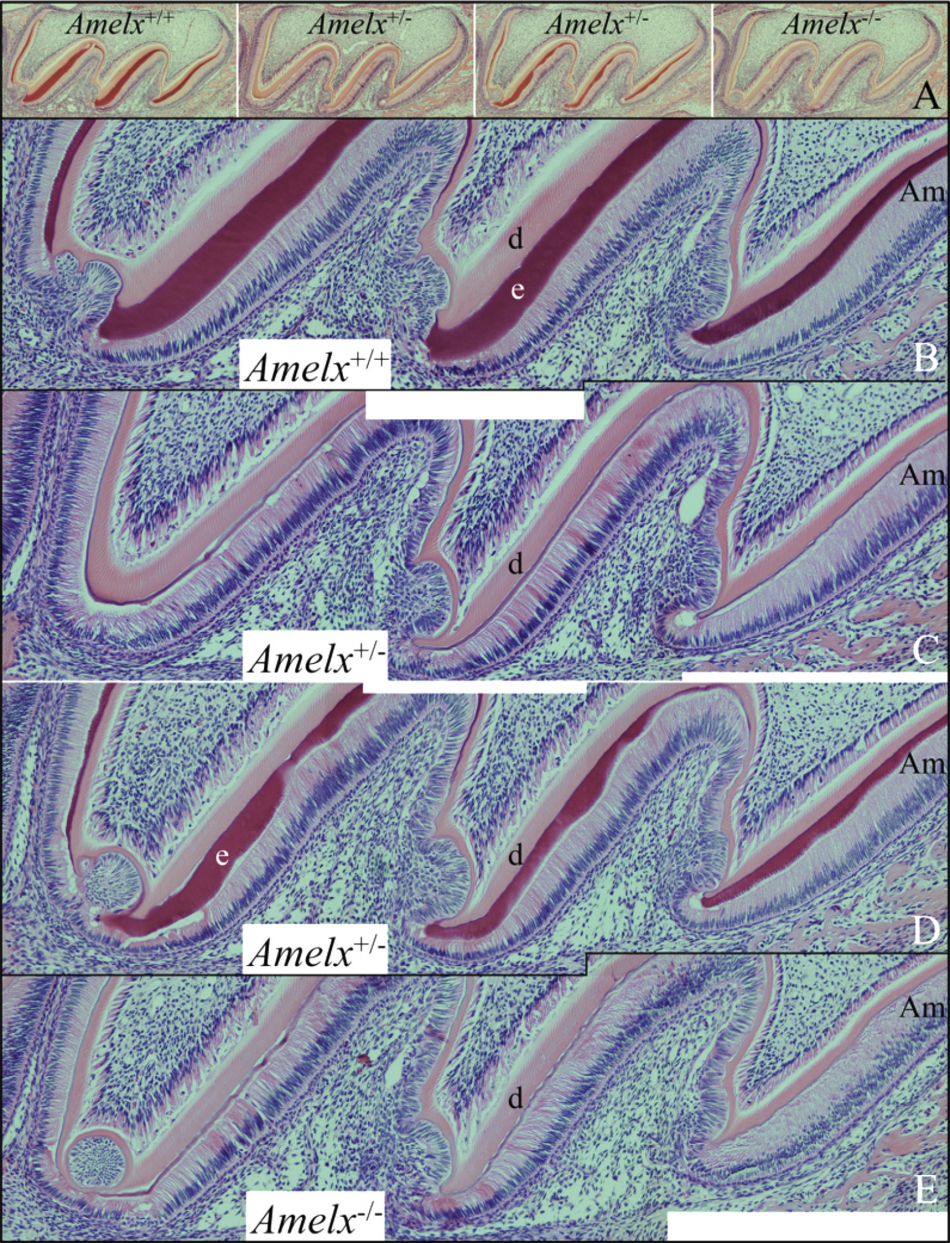
Maxillary D5 first molar histology. (A) Low‐magnification sections of four molars predominantly in the secretory stage of enamel formation. (B–E) Collages of high‐magnification images showing *Amelx* ^+/+^, *Amelx* ^+/*−*^, and *Amelx* ^*−*/*−*^ molar secretory‐stage ameloblasts. Note the variability in enamel thickness in the *Amelx* ^+/*−*^ molars. The heterozygous enamel in Panel C is virtually as thin as that of the null, whereas the heterozygous enamel in Panel D varies from being as thin as the null and as thick as the wild‐type enamel. Key: Am, ameloblasts; d, dentin; e, enamel.

**Figure 11:**
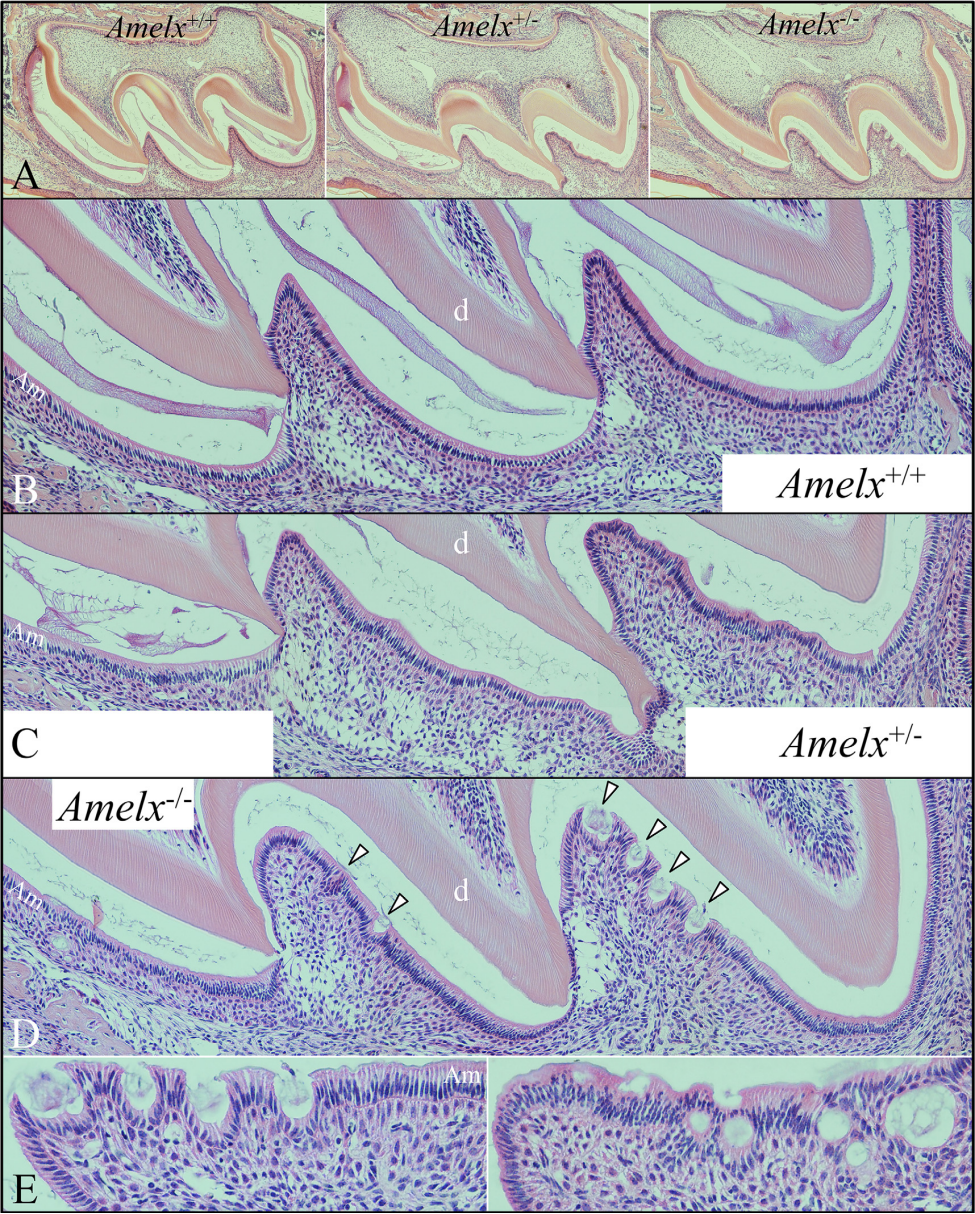
Maxillary D11 first molar histology. (A) Low‐magnification sections of four molars predominantly in the maturation stage of enamel formation. (B–D) Collages of high‐magnification images showing *Amelx* ^+/+^, *Amelx* ^+/*−*^, and *Amelx* ^*−*/*−*^ molar maturation‐stage ameloblasts. Note the apparent buckling of the ameloblast layer in the *Amelx* ^*−*/*−*^ molars (arrowheads). (E) Higher‐magnification images of nodules forming in and under the ameloblast layer. Organic matrix (or cell debris) within the nodules suggests difficulty in reabsorbing the amelogenin‐less matrix. Key: Am, ameloblasts; d, dentin; e, enamel.

The crowns of mandibular first molars were characterized by SEM on D14, immediately prior to their eruption into the oral cavity (Fig. 9). The *Amelx* ^+/+^ molar enamel was smooth and well‐contoured. The *Amelx* ^+/*−*^ enamel varied in thickness throughout. The *Amelx* ^+/*−*^ and *Amelx* ^*−*/*−*^ molar enamel exhibited thin cusps and numerous protruding mineral nodules, particularly on the occlusal surface. No histological evidence of nodule formation was evident in D5 developing first molars, where the ameloblasts were in the secretory stage (Fig. 10). The enamel that formed in D5 *Amelx* ^+/*−*^ molars varied in thickness. In some cases virtually all of the enamel was as thin as that formed in *Amelx* ^*−*/*−*^ molars (Fig. 10C). In other cases the enamel layer varied in thickness (Fig. 10D). Overall, no obvious cellular pathology was observed in the *Amelx* ^+/+^, *Amelx* ^+/*−*^, or *Amelx* ^*−*/*−*^ secretory‐stage ameloblasts. In contrast, there was clear histological evidence of nodule formation in D11 developing first molars, where the ameloblasts were in the maturation stage (Fig. 11). The ameloblast layer, which normally stretches over an expanding enamel surface as the enamel layer thickens, appeared to have buckled or folded around cell debris on the enamel surface or within the ameloblasts layer. This extracellular material mineralized to form pathological nodules that underwent rapid attrition following eruption of the molars.

## Discussion

Enamel is the distinctive, highly mineralized product of epithelial cells that covers the crowns of teeth and is called ganoine when covering the scales of fish. It contrasts with enameloid, the collagen‐based mineralized product of mesenchymal cells that is also found in fish teeth and scales. As enamel is produced by both the coelacanth and gar, its evolutionary origin must have preceded the split between Actinopterygii and Sarcopterygii about 450 million years ago (Ma) (Blair and Hedges 2005). Ganoine and enamel formation share the following similarities: both are deposited as thin mineral ribbons that elongate along an epithelial membrane, and are oriented to it. These ribbons initiate in close association with collagen fibers deposited by underlying osteoblasts or odontoblasts (Sire 1995). The onset of epithelial‐derived mineralization follows progressive disappearance of the basal lamina (Sire et al. 1987). After the mineral layer reaches its final thickness, it hypermineralizes through a maturation process that includes the progressive disappearance of organic matrix (Sire 1994). Because of these striking similarities in its structure and epithelial mechanism of formation, it was concluded that “ganoine is enamel” (Sire 1994). Both enameloid and enamel cover the crowns of gar teeth, with the enamel localizing to the collar region (Prostak et al. 1989).

Three SCPP genes/proteins are required for normal appositional growth of dental enamel: amelogenin (*Amelx*), enamelin (*Enam*), and ameloblastin (*Ambn*). These genes are found in the genomes of Coelacanth, lungfish, and in tetrapods that make dental enamel (Kawasaki and Amemiya 2014), but are generally absent or only marginally recognizable in teleosts, which make enameloid. Teleosts are ray‐finned fish that make up the vast majority of all fish species. The gar is a nonteleost actinopterygian that makes enameloid, enamel, and ganoine. Recently, *Enam* and *Ambn*, but not *Amel*, were identified in the Spotted Gar genome (Qu et al. 2015; Braasch et al. 2016), so amelogenin is not required to make enamel/ganoine in the gar. *Amel* is thought to have arisen through a duplication of *Ambn*, which in turn was spawned by a duplication of *Enam* (Sire et al. 2006), so the amelogenin gene is the youngest of the three SCPP genes (*Enam*,* Ambn*, and *Amel*). It is not known if *Amel* existed at the time of the Actinopterygii/Sarcopterygii divergence and was deleted in the line to gar, or if *Amel* arose later, in early sarcopterygians. Within Actinopterygii, the Holostei (including gars) split from the line to teleosts about 360 Ma (Near et al. 2012). Enamel/ganoine formation, along with *Enam* and *Ambn* must have been deleted in the line leading to teleosts soon after this split, as all teleosts lack them.

In this study we investigated the role of amelogenin in dental enamel formation through extensive characterization of the enamel formed in *Amelx* null mice. TEM images showed, for the first time, that *Amelx* ^*−*/*−*^ mice are able to generate the thin mineral ribbons that are a hallmark feature of true enamel. In contrast, *Enam* ^*−*/*−*^ mice were not able to generate enamel ribbons. Thus, the production of enamel mineral ribbons in mice requires *Enam*, but not *Amelx*. This finding is consistent with the previous observation that amelogenin is relatively less concentrated at the mineralization front where the enamel ribbons grow in length (Nanci et al. 1998).

In the *Amelx* null condition the mineral ribbons appear to fuse at some distance away from the ameloblast, while the unfused superficial extensions radiate toward the ameloblast membrane as mineralized fan‐like structures that continue to elongate at the mineralization front. The apparent fusion of mineral ribbons in *Amelx* ^*−*/*−*^ enamel supports Fincham's hypothesis that amelogenin separates and supports the secretory‐stage enamel ribbons (Fincham and Simmer 1997). The data also support the conclusion that enamelin is necessary to shape the early mineral ribbons, whereas amelogenin is not.

Ameloblasts in mammals have two, partially separate secretory surfaces associated with elaborating enamel ribbons: the interrod growth sites (IGS) and the rod growth sites (RGS) (Kallenbach 1973; Leblond and Warshawsky 1979; Nanci and Warshawsky 1984). The interrod growth site is located along the apical surface of the cells at their junction with adjacent ameloblasts, while rod enamel is produced by the Tomes’ process, a cytoplasmic extension distal to the apical membrane (Nanci and Warshawsky 1984). Both growth sites are characterized by irregular infoldings of the cell membrane, whereas the nonsecretory membrane that partially separates the sites is relatively smooth (Kallenbach 1973). The crystallites in each rod are deposited by a single Tomes’ process and are generated by a single ameloblast (Skobe 2006). The Tomes’ process of rodent ameloblasts penetrates about 18 *μ*m into the enamel layer (Risnes et al. 2002). The Tomes’ process does not form by forcing its way into the existing mineral layer; it develops following the formation of ~4 *μ*m of initial enamel, which is not organized into rod and interrod structures. The Tomes’ process becomes defined by the relatively rapid extension of the initial enamel ribbons by the incipient interrod growth sites at the borders of adjacent ameloblasts. The ameloblasts in *Amelx* ^*−*/*−*^ mice do not form a Tomes’ process. As the accumulated appositional growth over the entire secretory stage in the *Amelx* ^*−*/*−*^ mouse is only ~20 *μ*m, Tomes’ processes might not be able to form because the interrod growth sites cannot extend the interrod enamel sufficiently to define them. Indeed, amelogenin localization is normally higher on the raised interrod matrix between the Tomes’ process pits than in the pits themselves (Herold et al. 1987; Nanci et al. 1998), so amelogenin concentrates somewhat in the matrix area that must expand to form a Tomes’ process.

Reptilian enamel formation is characterized by a relatively flat mineralizing front with perpendicular crystallite orientation throughout its development (Boyde 1967). Tomes’ processes and the prismatic organization of enamel crystallites arose relatively recently during evolution, in mammals before the divergence of marsupials and placental mammals (Gasse et al. 2015), about 160 Ma (Luo et al. 2011). This was long after *Amel* was introduced during evolution and occurred at a time when amelogenin expression patterns were unchanging (Assaraf‐Weill et al. 2013; Gasse et al. 2015). It seems that failure to form a Tomes’ process in *Amelx* ^*−*/*−*^ mice is more likely to be due to the reduced matrix expansion that occurs in the absence of abundant amelogenin secretion, rather than by being directly caused by an absence of amelogenin. Amelogenin normally comprises about 90% of the secretory‐stage enamel matrix (Fincham et al. 1999) and in its absence the enamel layer does not expand properly, causing downstream consequences.

Rod and interrod enamel are both comprised of characteristic enamel ribbons, but because they elongate at different growth sites on the ameloblast distal membrane, they differ in their orientations (direction of growth) (Simmer and Fincham 1995; Moinichen et al. 1996). The rod represents the fossilized path traced out by the Tomes’ processes of the ameloblasts during enamel secretion (Boyde 1967). In the absence of amelogenin, modification in the shape of the mineralizing front following deposition of the initial enamel fails. The normal repetitive pattern of change in crystal orientation associated with rod and interrod structures is not observed. There are, however, localized independently mineralizing structures suggestive of rods that appear to be the pathological sequellae of sustained secretory‐stage appositional growth without amelogenin or proper thickening of the matrix.

The thickness of the enamel layer formed in *Amelx* ^+/*−*^ mice was highly variable, an effect believed to be caused by lyonization (Witkop 1967). During the early blastocyst stage, one of the two X chromosomes in a female cell is inactivated, equalizing the expression of genes with the single X chromosome of male cells (Gartler and Riggs 1983). With only one *Amelx* gene knocked out, female heterozygotes (*Amelx* ^+/*−*^) are mosaics of ameloblasts that either express normal amelogenin at normal levels or no amelogenin at all. The sheet of ameloblasts‐producing enamel is therefore comprised of cohorts of ameloblasts, each cohort descended from a single progenitor cell that expanded via cell division during odontogenesis. The *Amelx* ^+/*−*^ enamel layer varies in thickness in patterns that are unique to each tooth formed (Appendices S14–S18). The *Amelx* ^+/*−*^ enamel is thinnest (~20 *μ*m) where multiple cohorts of ameloblasts expressed only the *Amelx* knockout gene and thickest (>100 *μ*m) where multiple cohorts of ameloblasts expressed only wild‐type *Amelx*. In the incisors, where alternating rows of ameloblasts normally migrate laterally in opposite directions (and thereby generate an X‐shaped or decussation pattern of enamel rods), the rows of cells are sometimes compelled to ascend or descend steeply, and this may have pathological consequences that explain the increase in nodule formation observed in these areas.

We were surprised to observe extensive nodules on the *Amelx* ^+/*−*^ and *Amelx* ^*−*/*−*^ molars, although the enamel surface of these mice had been previously described as “rough and knobby” (Gibson et al. 2001). Our histological analyses of D5 and D11 first molars (Figs 9 and 10) suggested that the surface nodules formed during the maturation stage. This was surprising because this is after *Amelx* expression is downregulated (Wakida et al. 1999), but when residual amelogenin cleavage products are still being reabsorbed from the matrix. Perhaps the maturation‐stage ameloblast layer buckles due to there being too many cells covering too small of an enamel surface area. Alternatively, the nodules may simply be the manifestation of aberrant processes that become increasingly pathological with time.

There is broad consensus that mature dental enamel is comprised of crystallites very similar to hexagonal calcium hydroxyapatite [Ca_10_(PO_4_)_6_(OH)_2_; HAP] that are oriented with respect to their c‐axes (long axes) in the direction of the rods, but randomly oriented in their a‐axes perpendicular to the plane of the c‐direction (Glas 1962). To our knowledge, this is the first time that X‐rays have been focused on dental enamel and produced an octacalcium phosphate [Ca_8_H_2_(PO_4_)_6_; OCP] diffraction pattern. In vitro, when low supersaturation calcium phosphate solutions that were supersaturated only with respect to tricalcium phosphate [Ca_3_(PO_4_)_2_; TCP] or hydroxyapatite were seeded with natural enamel crystals, the crystals grew by the continued addition of HAP; however, if high supersaturated solutions supersaturated with respect to dicalcium phosphate dihydrate [CaHPO_4_·2H_2_O; DCPD], OCP and TCP were seeded with enamel crystals, the crystals grew by the addition of OCP platelets that slowly converted into HAP (Tomazic et al. 1976). Growth of the OCP intermediate phase on HAP at high supersaturations was inhibited by magnesium (Tomazic et al. 1975). OCP growth is favored over HAP at lower Ca/P ratios and decreasing pH (Meyer and Eanes 1978). In the *Amelx* ^*−/−*^ mice, the enamel layer is mineralizing at a greatly reduced rate relative to the wild type. By the end of the secretory stage, the *Amelx* ^*−/−*^ enamel is only a sixth as thick and less than half as hard as normal enamel. Because of the reduced deposition of calcium and phosphate into the mineral phase, the concentration of these ions in the matrix might rise to the point where OCP is favored and then forms on top of existing HAP crystals.

Long ago it was proposed that the initial mineral phase in enamel is octacalcium phosphate (Brown 1965; Simmer and Fincham 1995). However, subsequent studies could find no evidence of octacalcium phosphate in embryonic bovine enamel (Landis and Navarro 1983; Landis et al. 1984, 1988). The initial mineral in enamel is currently believed to be a transient amorphous calcium phosphate (ACP) phase that converts into HAP. This is held because the enamel mineral ribbons fail to show a crystalline electron diffraction pattern (Beniash et al. 2009). Full‐length amelogenin (P173) (Wiedemann‐Bidlack et al. 2011) and its major proteolytic cleavage product (P148) (Kwak et al. 2009), as well as full‐length LRAP (P56) (Le Norcy et al. 2011a) and its major cleavage product (P40) (Le Norcy et al. 2011b) stabilize amorphous calcium phosphate for extended periods of time in vitro. Perhaps amelogenin plays a role in regulating the transition from ACP to HAP so as to avoid the formation of OCP.

Amelogenin is almost synonymous with dental enamel formation. It is the most abundant protein in developing enamel and was the first enamel protein to be characterized by protein sequencing (Fincham et al. 1981; Takagi et al. 1984), the first to have its cDNA cloned (Snead et al. 1983) and characterized (Snead et al. 1985), and the first to be expressed in recombinant form (Simmer et al. 1994b). *AMELX* was the first gene shown to cause amelogenesis imperfecta when defective (Lagerström et al. 1991), and mouse *Amelx* was the first enamel gene to be knocked out (Gibson et al. 2001). This examination of enamel formed C57BL/6 *Amelx* ^+/+^, *Amelx* ^+/*−*^, and *Amelx* ^*−*/*−*^ mice in the light of current knowledge provides a fresh insight into dental enamel formation and amelogenin's role in it.

Amelogenin is secreted even before the first enamel ribbons form; however, the thin enamel ribbons still form in its absence. Amelogenin does not initiate or shape the enamel ribbons, but in its absence the ribbons fuse and grow into thin plates of octacalcium phosphate, so they do adopt a different shape. A key function of amelogenin is to form a gel matrix that separates and supports the mineral ribbons. Amelogenin does not lengthen the mineral ribbons, but its continued secretion and accumulation expands the extracellular matrix, which is necessary for sustained crystal elongation. Amelogenin does not form the Tomes’ process, but its ability to expand the matrix at interrod growth sites is necessary to define it as a cell process. Amelogenin does not orient the enamel ribbons, this is done by the rod and interrod growth sites, but without amelogenin the architecture of these sites cannot be established. Rod and interrod organization of the enamel ribbons fails. Without amelogenin the enamel layer only reaches one sixth of its normal thickness and even that layer shows reduced mineral density and hardness. Whatever influence amelogenins have in determining the final mineral phase, it is lost in the knockout. With only a fraction of the normal mineral forming, extracellular ion concentrations likely rise and amelogenesis degenerates into an increasingly pathological process that forms plates of OCP and mineralized surface nodules.

## Conflict of Interest

None declared.

## Supporting information


**Appendix S1.** Reported *AMELX* disease‐causing mutations.
**Appendix S2.** Oral photos of 7‐week wild‐type (*Amelx* ^+/+^) mouse.
**Appendix S3.** Western blot and RT‐PCR analyses of amelogenin expression.
**Appendix S4.** *Amelx* ^*−*/*−*^ (#7) mandibular incisor histology at 7 weeks.
**Appendix S5.** *Amelx* ^+/*−*^ (#11) mandibular incisor histology at 7 weeks.
**Appendix S6.** *Amelx* ^+/*−*^ (#13) mandibular incisor histology at 7 weeks.
**Appendix S7.** *Amelx* ^+/*−*^ (#15) mandibular incisor histology at 7 weeks.
**Appendix S8.** *Amelx* ^+/*−*^ (#17) mandibular incisor histology at 7 weeks.
**Appendix S9.** SEM images of molar roots.
**Appendix S10.** Backscatter electron microscopy of incisor cross sections of *Amelx* ^*−*/*−*^ mouse 732.
**Appendix S11.** Backscatter electron microscopy of incisor cross sections of *Amelx* ^*−*/*−*^ mouse 733.
**Appendix S12.** Backscatter electron microscopy of incisor cross sections of *Amelx* ^*−*/*−*^ mouse 774.
**Appendix S13.** Backscatter electron microscopy of incisor cross sections of *Amelx* ^*−*/*−*^ mouse 780.
**Appendix S14.** Backscatter electron microscopy of incisor cross sections of *Amelx* ^+/*−*^ mouse 771.
**Appendix S15.** Backscatter electron microscopy of incisor cross sections of *Amelx* ^+/*−*^ mouse 787.
**Appendix S16.** Backscatter electron microscopy of incisor cross sections of *Amelx* ^+/*−*^ mouse 797.
**Appendix S17.** Backscatter electron microscopy of incisor cross sections of *Amelx* ^+/*−*^ mouse 799.
**Appendix S18.** Backscatter electron microscopy of incisor cross sections of *Amelx* ^+/*−*^ mouse 802.
**Appendix S19.** Backscatter electron microscopy of incisor cross sections of *Amelx* ^+/+^ mouse 35.
**Appendix S20.** Backscatter electron microscopy images for enamel thickness.
**Appendix S21.** *Amelx* ^+/*−*^ nanohardness testing.
