# Supplementary material for: Enamel ribbons, surface nodules, and octacalcium phosphate in C57BL/6 Amelx −/− mice and Amelx +/− lyonization
Source: Mol Genet Genomic Med. 2016 Oct 5;4(6):641–61. doi: 10.1002/mgg3.252 (PMC5118209; doi:10.1002/mgg3.252)
Supplement: Supplementary file 1 — Appendix S1. Reported AMELX disease‐causing mutations. Appendix S2. Oral photos of 7‐week wild‐type (Amelx +/+) mouse. Appendix S3. Western blot and RT‐PCR analyses of amelogenin expression. Appendix S4. Amelx −/− (#7) mandibular incisor histology at 7 weeks. Appendix S5. Amelx +/− (#11) mandibular incisor histology at 7 weeks. Appendix S6. Amelx +/− (#13) mandibular incisor histology at 7 weeks. Appendix S7. Amelx +/− (#15) mandibular incisor histology at 7 weeks. Appendix S8. Amelx +/− (#17) mandibular incisor histology at 7 weeks. Appendix S9. SEM images of molar roots. Appendix S10. Backscatter electron microscopy of incisor cross sections of Amelx −/− mouse 732. Appendix S11. Backscatter electron microscopy of incisor cross sections of Amelx −/− mouse 733. Appendix S12. Backscatter electron microscopy of incisor cross sections of Amelx −/− mouse 774. Appendix S13. Backscatter electron microscopy of incisor cross sections of Amelx −/− mouse 780. Appendix S14. Backscatter electron microscopy of incisor cross sections of Amelx +/− mouse 771. Appendix S15. Backscatter electron microscopy of incisor cross sections of Amelx +/− mouse 787. Appendix S16. Backscatter electron microscopy of incisor cross sections of Amelx +/− mouse 797. Appendix S17. Backscatter electron microscopy of incisor cross sections of Amelx +/− mouse 799. Appendix S18. Backscatter electron microscopy of incisor cross sections of Amelx +/− mouse 802. Appendix S19. Backscatter electron microscopy of incisor cross sections of Amelx +/+ mouse 35. Appendix S20. Backscatter electron microscopy images for enamel thickness. Appendix S21. Amelx +/− nanohardness testing. [file MGG3-4-641-s001.pdf]

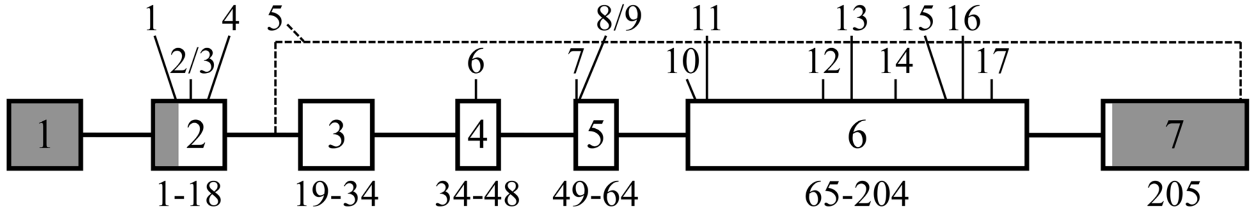

#	Location	Gene	cDNA	Protein	References
1	Exon 1	g.6378T>C	c.2T>C	p.Met1Thr (or p.?)	(1)
2	1	g.6387G>C	c.11G>C	p.Trp4Ser	(1)
3	1	g.6387G>A	c.11G>A	p.Trp4*	(2)
4	1	g.6390_6398del9	c.14_22del9	p.Ile5_Ala8delinsThr	(3)
5	Intron 2 – Exon 7	g.7527_12249del4723	c.55-840_665del4723	p.0	(4, 5)
6	Exon 4	g.9708T>C	c.120T>C	p.Ala40Ala or p.=	(6)
7	Exon 5	g.9831C>T	c.152C>T	p.Thr51Ile	(7)
8	Exon 5	g.9834delC	c.155delC	p.Pro52Leufs*2	(8, 9)
9	Exon 5	g.9834C>G	c.155C>G	p.Pro52Arg	(10, 11)
10	Exon 6	g.10157C>A	c.208C>A	p.Pro70Thr	(11-15)
11	Exon 6	g.10179A>T	c.230A>T	p.His77Leu	(16)
12	Exon 6	g.10334delC	c.385delC	p.His129Thrfs*60	(17)
13	Exon 6	g.10369delC	c.420delC	p.Tyr141Thrfs*48	(18)
14	Exon 6	g.10422delC	c.473delC	p.Pro158Hisfs*31	(7, 11)
15	Exon 6	g.10467delC	c.518delC	p.Pro173Leufs*16	(19)
16	Exon 6	g.10490delC	c.541delC	p.Leu181Cysfs*8	(16, 20)
17	Exon 6	g.10520G>T	c.571G>T	p.Glu191*	(7)

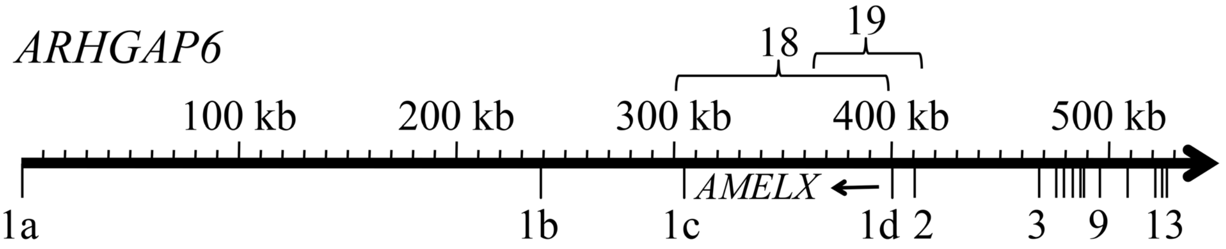

18	g.302534_398773del96240	no amelogenin	ARHGAP6
19	g.363924_416577del52654insA	no amelogenin	No ARHGAP6

S1 Appendix. Reported *AMELX* disease-causing mutations. ***Top:*** *AMELX* gene structure: numbered boxes are exons above the segment of amino acids encoded by it. Mutation 1 altered the *Amelx* translation initiation codon, so it is uncertain if any proteiin was translated. Mutation 5 is the deletion indicated by dashed lines. The clinical phenotype of mutation 5 was described before the mutation was identified (21, 22). Mutation 6 increased inclusion of Exon 4, which is normally skipped during splicing of the primary transcript. The gene numbers start from the first nucleotide of the *AMELX* reference sequence NG_012040.1. The cDNA numbers start from the translation initiation site of *AMELX* cDNA reference sequence NM_182680.1. ***Bottom:*** Deletions of *AMELX* in *ARHGAP6*. Mutation 18 was a 96240 bp deletion that included *ARHGAP6* promoter 1c and *AMELX*. Mutation 19 was a 52654 deletion that included *ARHGAP6* promoter 1d, *AMELX* and *ARHGAP6* Exon 2.

References for S1 Appendix

1. Kim J-W, Simmer JP, Hu YY, Lin BP-L, Boyd C, Wright JT, et al. Amelogenin p.M1T and p.W4S mutations underlying hypoplastic X-linked amelogenesis imperfecta. J Dent Res. 2004;83:378-83.
2. Sekiguchi H, Kiyoshi M, Yakushiji M. DNA diagnosis of X-linked amelogenesis imperfecta using PCR detection method of the human amelogenin gene. Dent Japan. 2001;37:109-12.
3. Lagerstrom-Fermer M, Nilsson M, Backman B, Salido E, Shapiro L, Pettersson U, et al. Amelogenin signal peptide mutation: correlation between mutations in the amelogenin gene (AMGX) and manifestations of X-linked amelogenesis imperfecta. Genomics. 1995;26(1):159-62.
4. Lagerström M, Dahl N, Iselius L, Bäckman B, Pettersson U. Mapping of the gene for X-linked *amelogenesis imperfecta* by linkage analysis. Am J Hum Genet. 1990;46(1):120-5.
5. Lagerström M, Dahl N, Nakahori Y, Nakagome Y, Backman B, Landegren U, et al. A deletion in the amelogenin gene (AMG) causes X-linked amelogenesis imperfecta (AIH1). Genomics. 1991;10(4):971-5.
6. Cho ES, Kim KJ, Lee KE, Lee EJ, Yun CY, Lee MJ, et al. Alteration of conserved alternative splicing in AMELX causes enamel defects. J Dent Res. 2014;93(10):980-7 LID - 10.1177/0022034514547272 [doi].
7. Lench NJ, Winter GB. Characterisation of molecular defects in X-linked amelogenesis imperfecta (AIH1). Hum Mutat. 1995;5(3):251-9.
8. Aldred MJ, Crawford PJ, Roberts E, Thomas NS. Identification of a nonsense mutation in the amelogenin gene (AMELX) in a family with X-linked amelogenesis imperfecta (AIH1). Hum Genet. 1992;90(4):413-6.
9. Lench NJ, Brook AH, Winter GB. SSCP detection of a nonsense mutation in exon 5 of the amelogenin gene (AMGX) causing X-linked amelogenesis imperfecta (AIH1). Hum Mol Genet. 1994;3(5):827-8.
10. Kida M, Sakiyama Y, Matsuda A, Takabayashi S, Ochi H, Sekiguchi H, et al. A novel missense mutation (p.P52R) in amelogenin gene causing X-linked amelogenesis imperfecta. J Dent Res. 2007;86(1):69-72.
11. Wright JT, Torain M, Long K, Seow K, Crawford P, Aldred MJ, et al. Amelogenesis imperfecta: genotype-phenotype studies in 71 families. Cells Tissues Organs. 2011;194(2-4):279-83.
12. Collier PM, Sauk JJ, Rosenbloom SJ, Yuan ZA, Gibson CW. An amelogenin gene defect associated with human X-linked amelogenesis imperfecta. Arch Oral Biol. 1997;42(3):235-42.
13. Hart S, Hart T, Gibson C, Wright JT. Mutational analysis of X-linked amelogenesis imperfecta in multiple families. Arch Oral Biol. 2000;45(1):79-86.
14. Ravassipour DB, Hart PS, Hart TC, Ritter AV, Yamauchi M, Gibson C, et al. Unique enamel phenotype associated with amelogenin gene (AMELX) codon 41 point mutation. J Dent Res. 2000;79(7):1476-81.
15. Chan HC, Estrella NM, Milkovich RN, Kim JW, Simmer JP, Hu JC. Target gene analyses of 39 amelogenesis imperfecta kindreds. Eur J Oral Sci. 2011;119(Suppl 1):311-23.
16. Hart PS, Aldred MJ, Crawford PJ, Wright NJ, Hart TC, Wright JT. Amelogenesis imperfecta phenotype-genotype correlations with two amelogenin gene mutations. Arch Oral Biol. 2002;47(4):261-5.
17. Sekiguchi H, Alaluusua S, Minaguchi K, Yakushiji M. A new mutation in the amelogenin gene causes X-linked amelogenesis imperfecta. J Dent Res. 2001;80(IADR Abstract 722):617.
18. Greene SR, Yuan ZA, Wright JT, Amjad H, Abrams WR, Buchanan JA, et al. A new frameshift mutation encoding a truncated amelogenin leads to X-linked amelogenesis imperfecta. Arch Oral Biol. 2002;47(3):211-7.
19. Lee KE, Lee SK, Jung SE, Song SJ, Cho SH, Lee ZH, et al. A novel mutation in the AMELX gene and multiple crown resorptions. Eur J Oral Sci. 2011;119 Suppl 1:324-8.
20. Kindelan SA, Brook AH, Gangemi L, Lench N, Wong FS, Fearne J, et al. Detection of a novel mutation in X-linked amelogenesis imperfecta. J Dent Res. 2000;79(12):1978-82.
21. Backman B, Holmgren G. Amelogenesis imperfecta: a genetic study. Hum Hered. 1988;38(4):189-206.
22. Backman B. Amelogenesis imperfecta--clinical manifestations in 51 families in a northern Swedish county. Scand J Dent Res. 1988;96(6):505-16.

Amelx$^{+/+}$

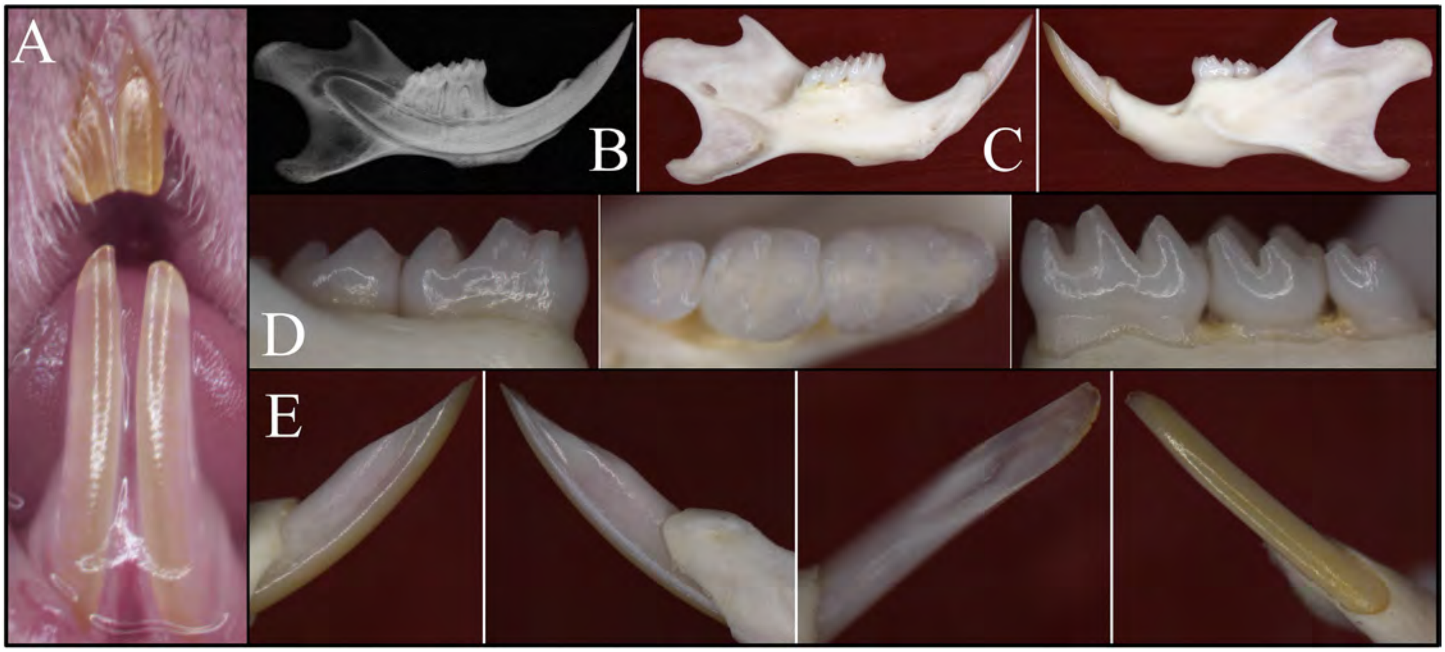

S2 Appendix. Oral photos of 7-week wild-type (*Amelx*$^{+/+}$) mouse.

A: Frontal view of incisors *in situ*.
B: Radiograph of right hemi-mandible.
C: Right and left hemi-mandibles following removal of soft tissues.
D: Buccal, occlusal, and lingual views of mandibular molars.
E: Lateral, mesial, lingual, facial views of a mandibular incisor.

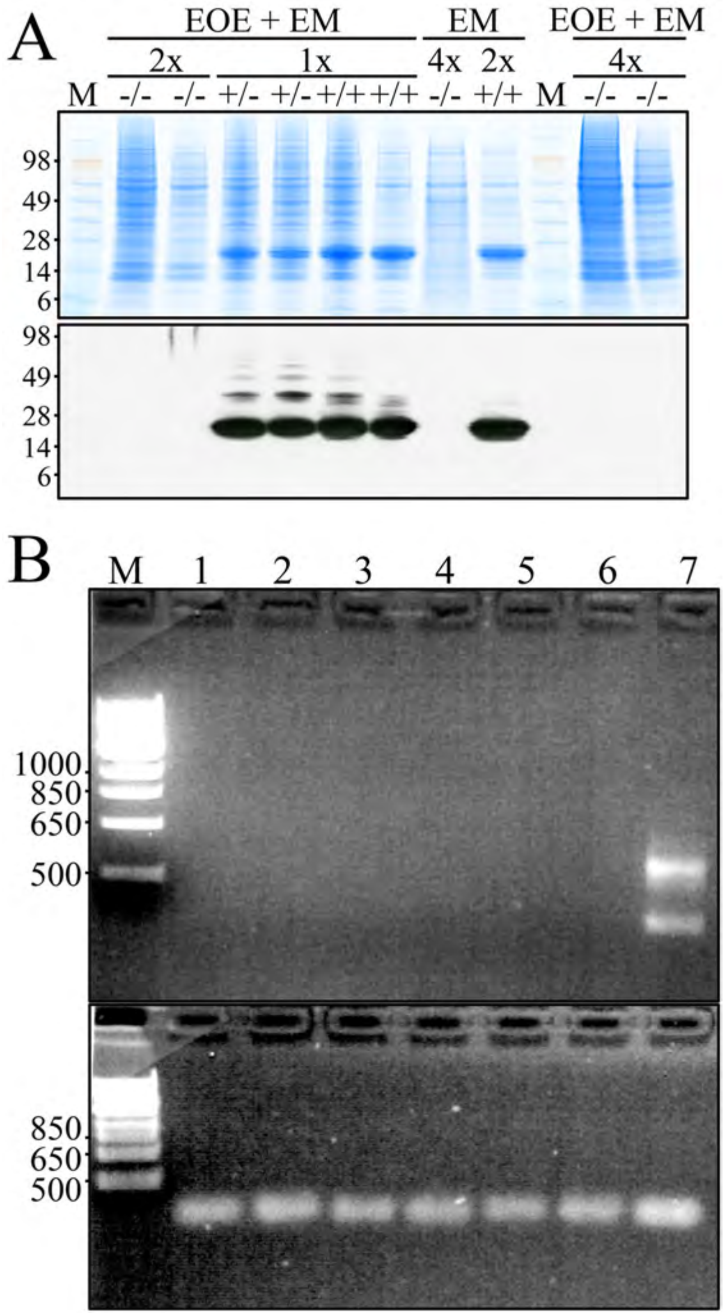

S3 Appendix. Western blot & RT-PCR analyses of amelogenin expression. ***A:*** SDS-PAGE and Western blot. Amelogenin protein was not detected in D5 first molars in *Amelx*$^{-/-}$ mice using a polyclonal antibody raised against recombinant mouse amelogenin (rM179). EOE: enamel organ epithelia; EM: enamel matrix. No amelogenin protein was detected in the enamel organ epithelia or enamel matrix of D5 first molars in *Amelx*$^{-/-}$ mice. ***B:*** Ethidium bromide-stained 1% agarose gels of amelogenin RT-PCR amplification products (top) and GAPDH (bottom) using RNA isolated from soft tissue surrounding the roots of 6 month-old first molars. M: 1 kb size standard; Lanes 1-4: *Amelx*$^{+/+}$ mice; Lanes 5-6: *Amelx*$^{-/-}$ mice (negative control); Lane 7 *Amelx*$^{+/+}$ D5 EOE (positive control). No amelogenin transcripts were observed in tissues surrounding the first molars of 6 month-old *Amelx*$^{+/+}$ mice. Control bands are predicted to be 579 bp for M180 and 216 bp for M59 (also known as the Leucine Rich Amelogenin Protein, or LRAP).

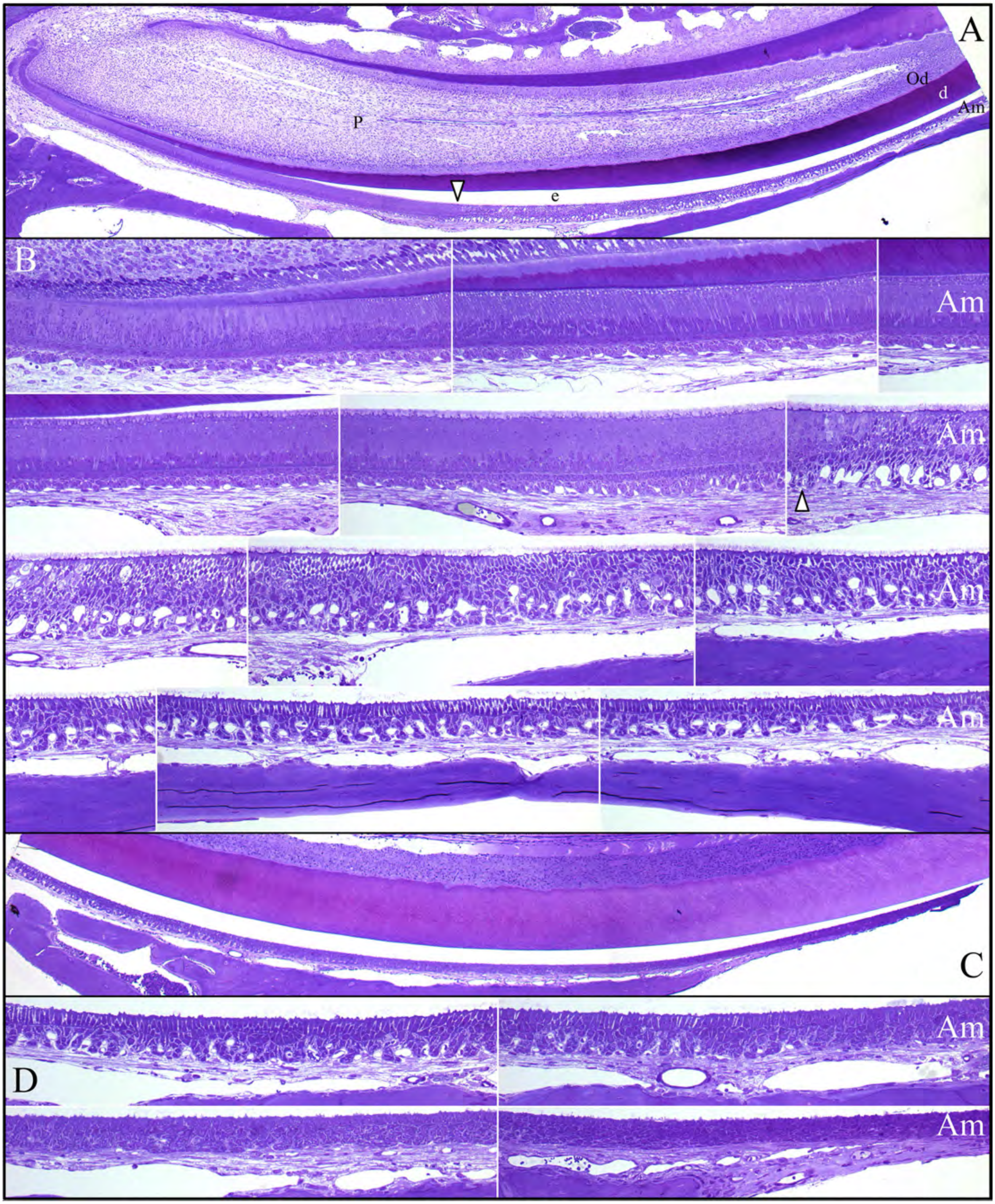

S4 Appendix. *Amelx*$^{-/-}$ (#7) mandibular incisor histology at 7-weeks. ***A:*** Low magnification of a longitudenal incisor section from the apical block. ***B:*** Higher magnification of ameloblast layer on apical block. ***C:*** Low magnification of longitudenal section from the incisal block. ***D:*** Higher magnification of the ameloblast layer from the incisal block. ***Key:*** Am, ameloblasts; d, dentin; e, enamel. Arrowheads mark the beginning of the transition from secretory to maturation stage.

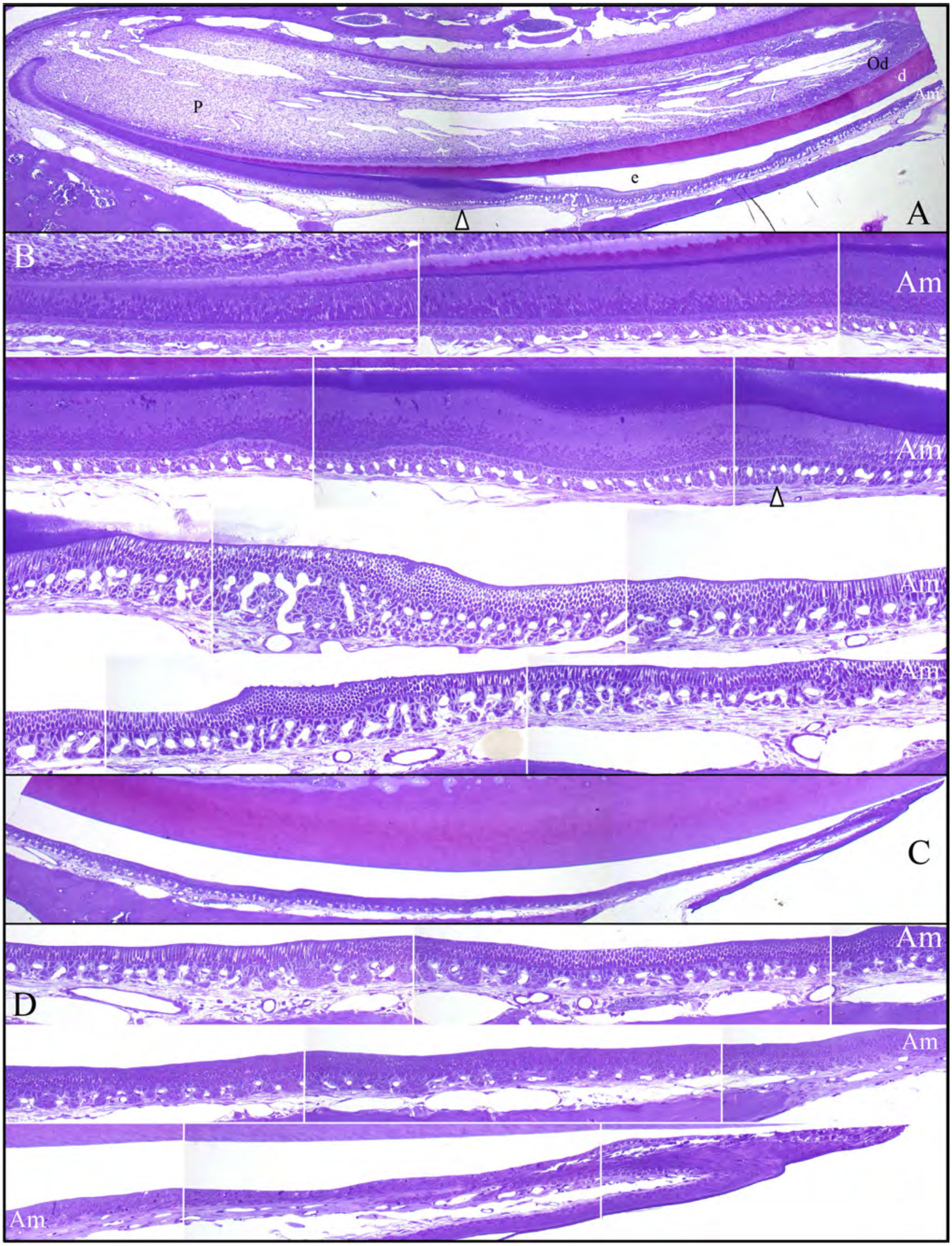

S5 Appendix. *Amelx*$^{+/-}$ (#11) mandibular incisor histology at 7-weeks. ***A:*** Low magnification of a longitudenal incisor section from the apical block. ***B:*** Higher magnification of ameloblast layer on apical block. ***C:*** Low magnification of longitudenal section from the incisal block. ***D:*** Higher magnification of the ameloblast layer from the incisal block. ***Key:*** Am, ameloblasts; d, dentin; e, enamel. Arrowheads mark the beginning of the transition from secretory to maturation stage.

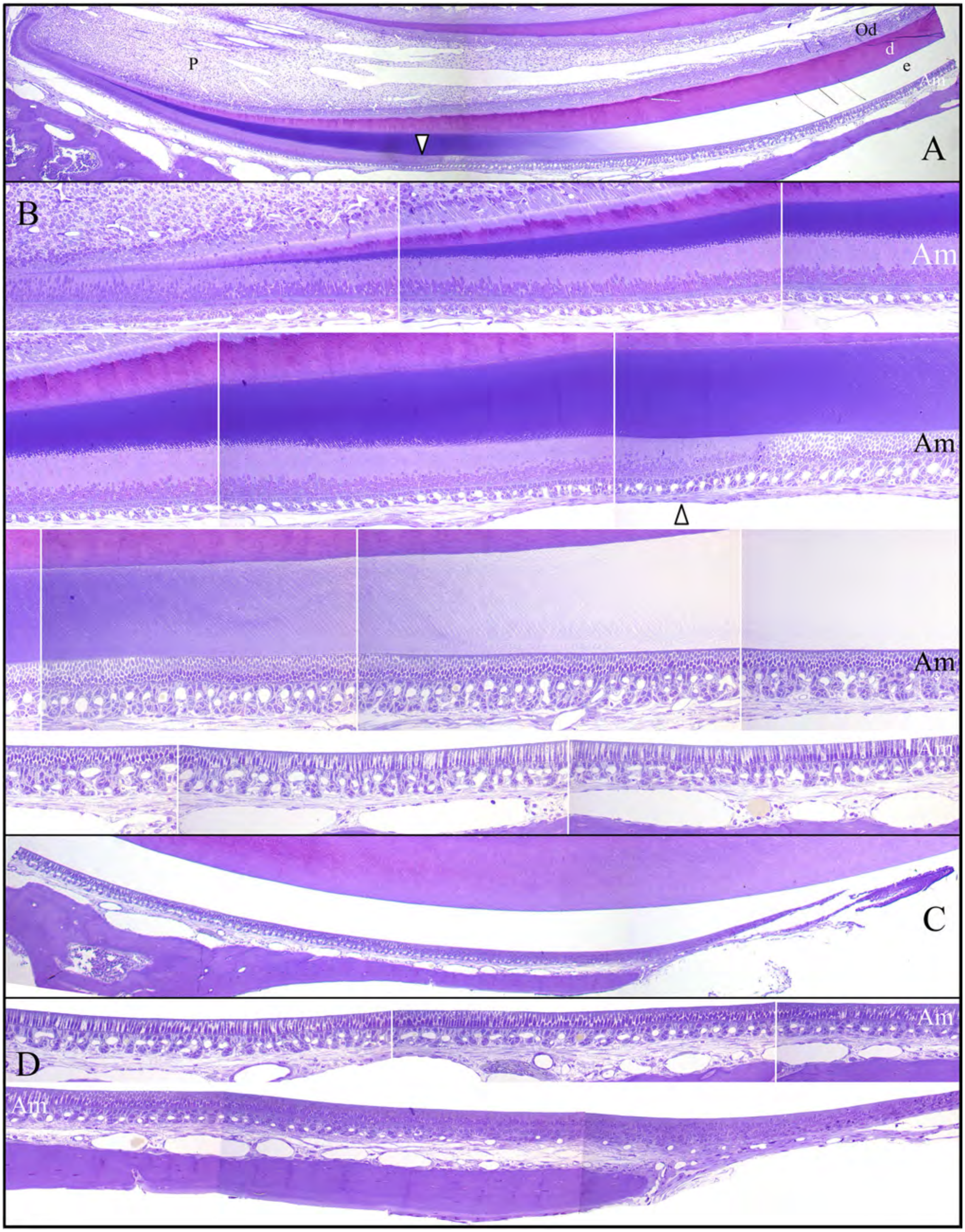

S6 Appendix. *Amelx*$^{+/-}$ (#13) mandibular incisor histology at 7-weeks. ***A:*** Low magnification of a longitudenal incisor section from the apical block. ***B:*** Higher magnification of ameloblast layer on apical block. ***C:*** Low magnification of longitudenal section from the incisal block. ***D:*** Higher magnification of the ameloblast layer from the incisal block. ***Key:*** Am, ameloblasts; d, dentin; e, enamel. Arrowheads mark the beginning of the transition from secretory to maturation stage.

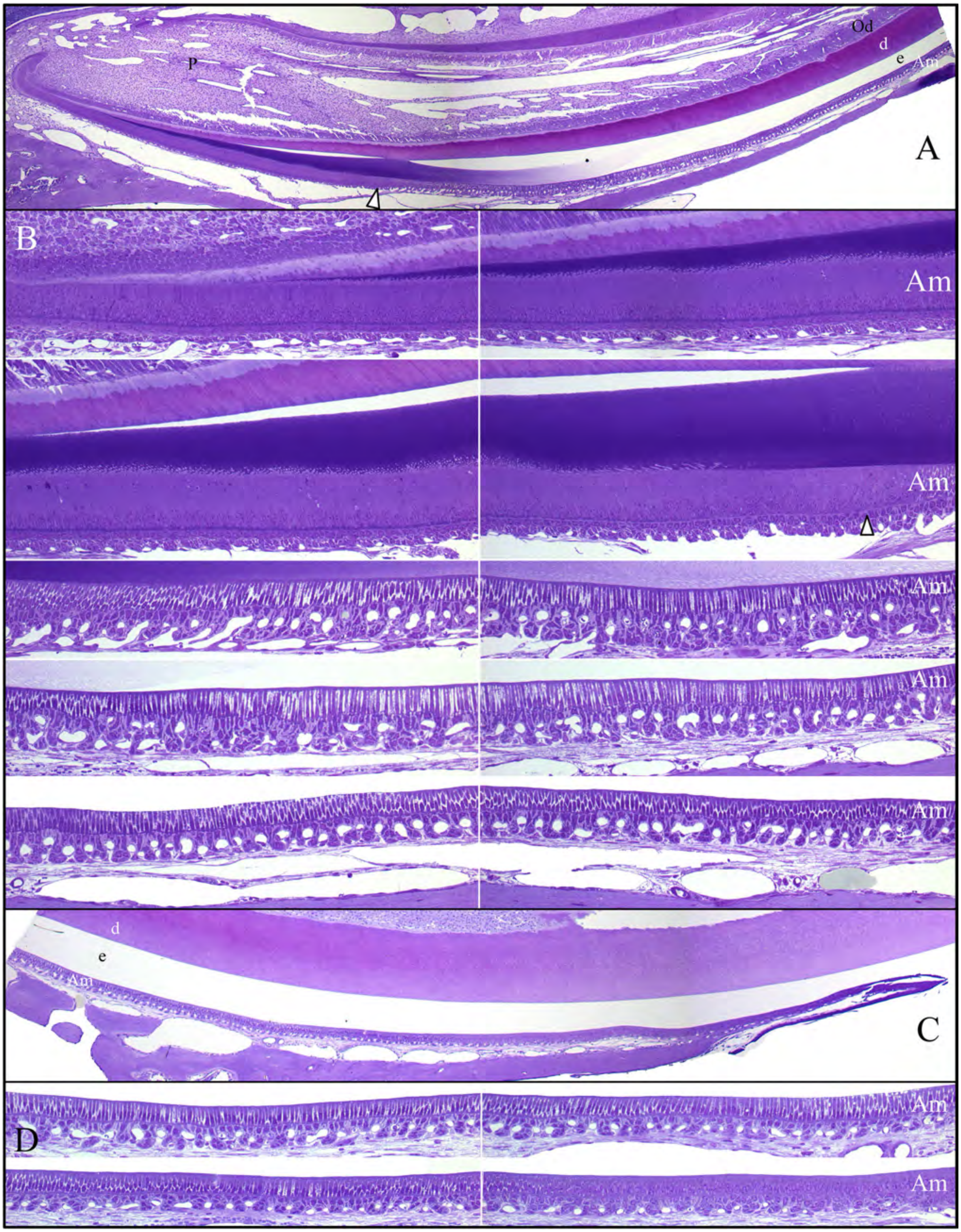

S7 Appendix. *Amelx*$^{+/-}$ (#15) mandibular incisor histology at 7-weeks. ***A:*** Low magnification of a longitudenal incisor section from the apical block. ***B:*** Higher magnification of ameloblast layer on apical block. ***C:*** Low magnification of longitudenal section from the incisal block. ***D:*** Higher magnification of the ameloblast layer from the incisal block. ***Key:*** Am, ameloblasts; d, dentin; e, enamel. Arrowheads mark the beginning of the transition from secretory to maturation stage.

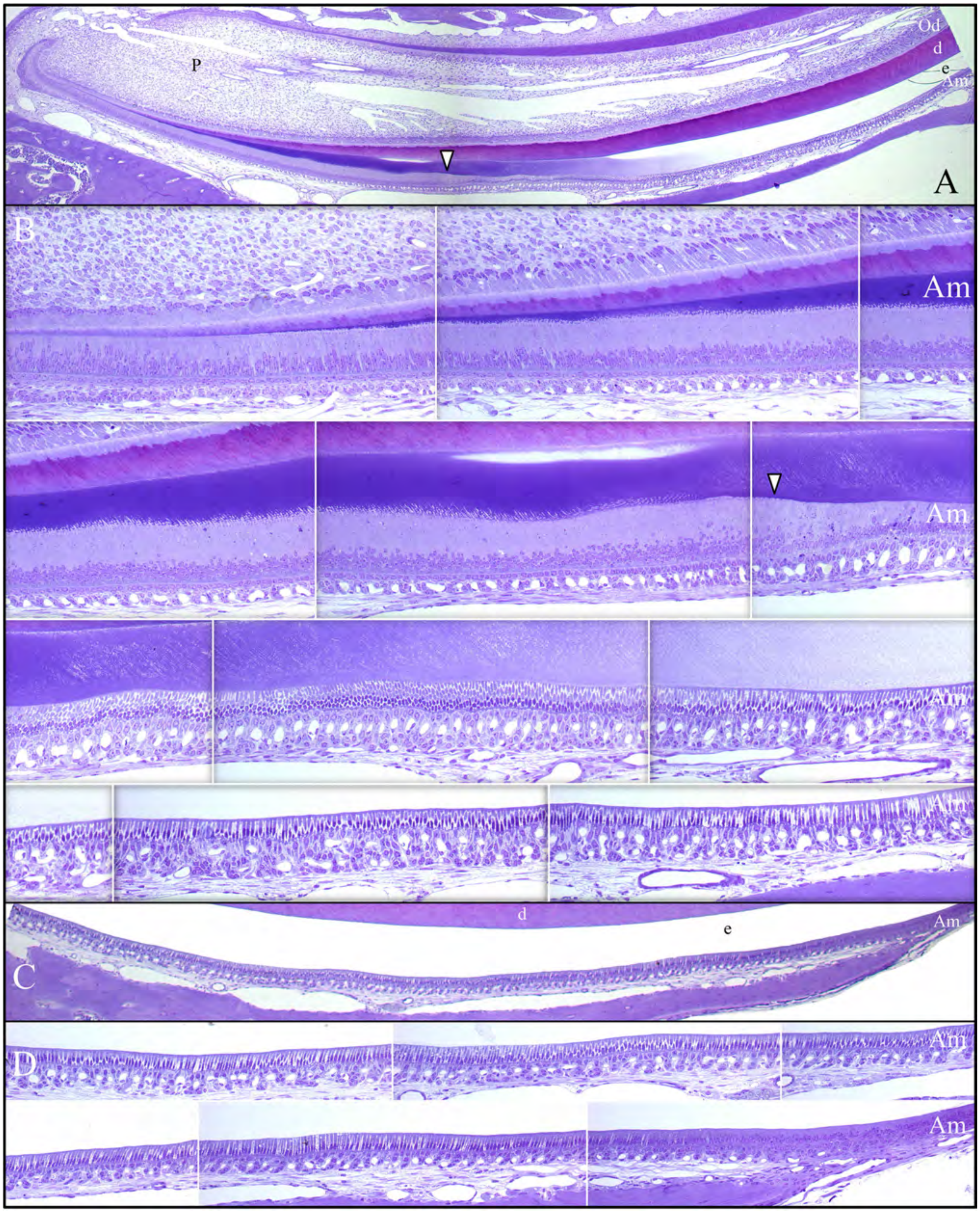

S8 Appendix. *Amelx*$^{+/-}$ (#17) mandibular incisor histology at 7-weeks. ***A:*** Low magnification of a longitudenal incisor section from the apical block. ***B:*** Higher magnification of ameloblast layer on apical block. ***C:*** Low magnification of longitudenal section from the incisal block. ***D:*** Higher magnification of the ameloblast layer from the incisal block. ***Key:*** Am, ameloblasts; d, dentin; e, enamel. Arrowheads mark the beginning of the transition from secretory to maturation stage.

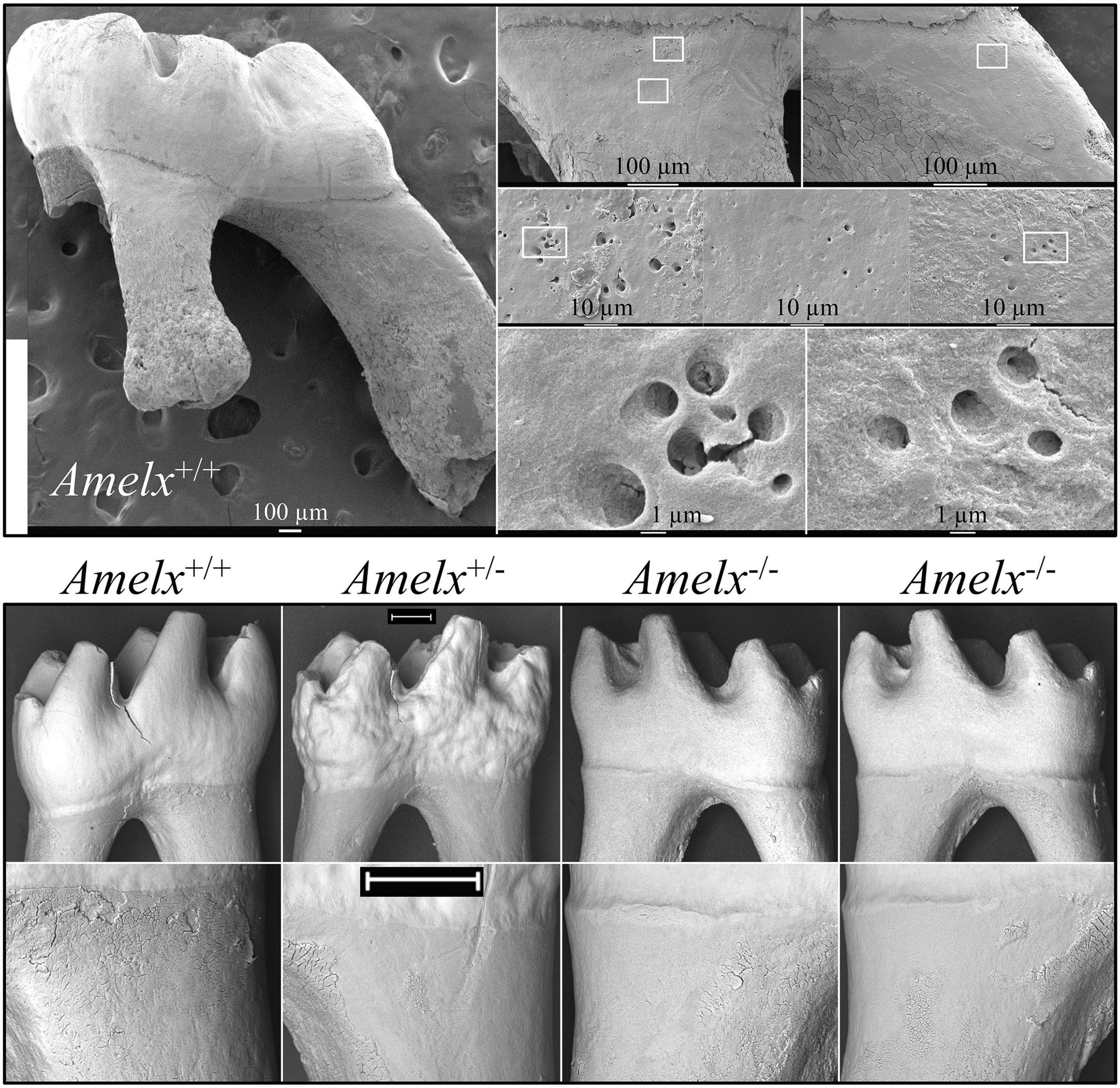

S9 Appendix. SEM images of molar roots. ***Top:*** *Amelx*$^{+/+}$ (wild-type) from F3 generation showed root resorption that diminished or disappeared with continued out breeding with C56Bl/6 mice. ***Bottom:*** F7 generation in C56Bl/6 background showed no apparent differences in their roots among the *Amelx*$^{+/+}$, *Amelx*$^{+/-}$, and *Amelx*$^{-/-}$ molars. Size bars = 200 µm.

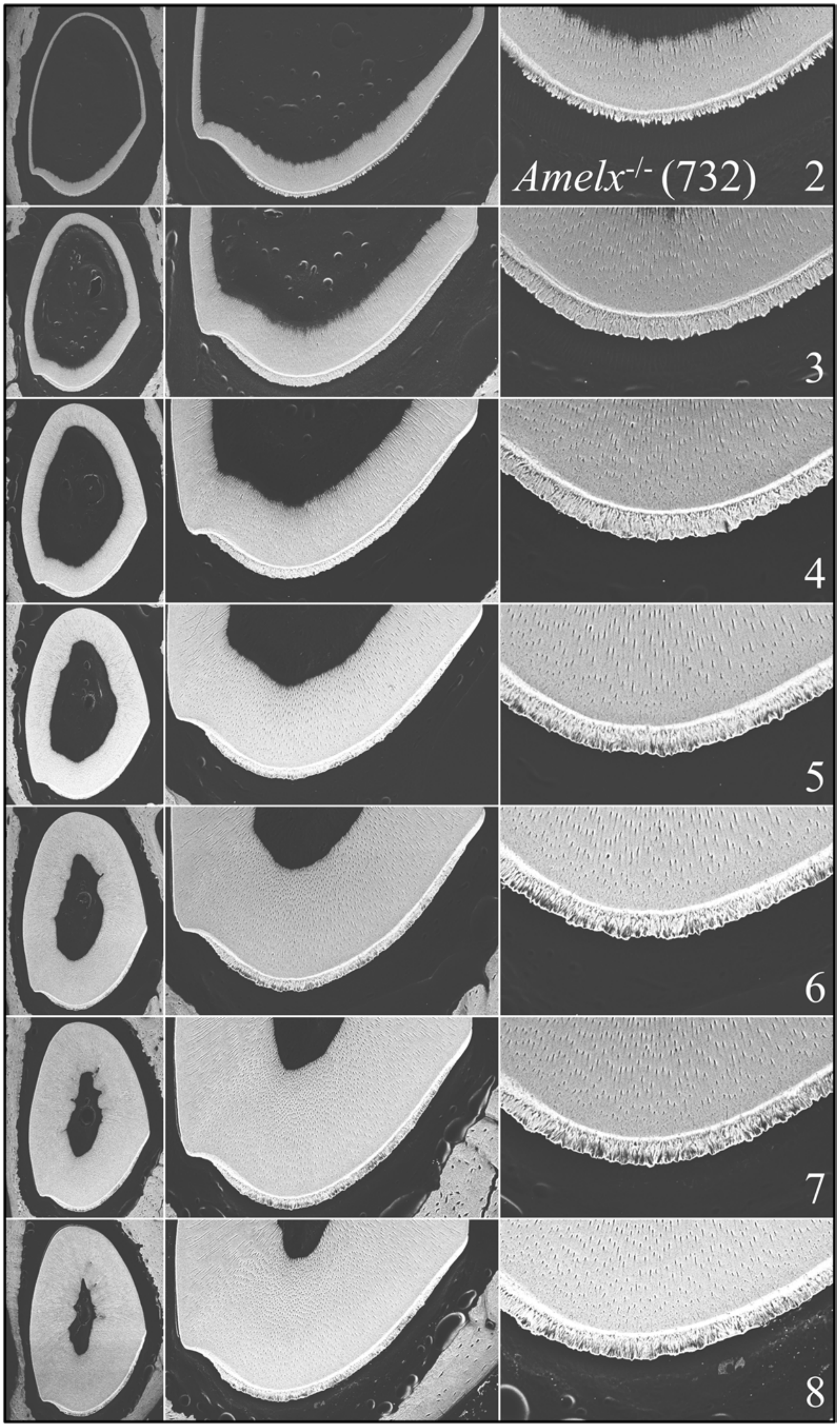

S10 Appendix. Backscatter electron microscopy of successive cross sections from 1 mm increments starting basally (top) and moving incisally (bottom) along the 7-week mandibular incisor of *Amelx*$^{-/-}$ mouse 732.

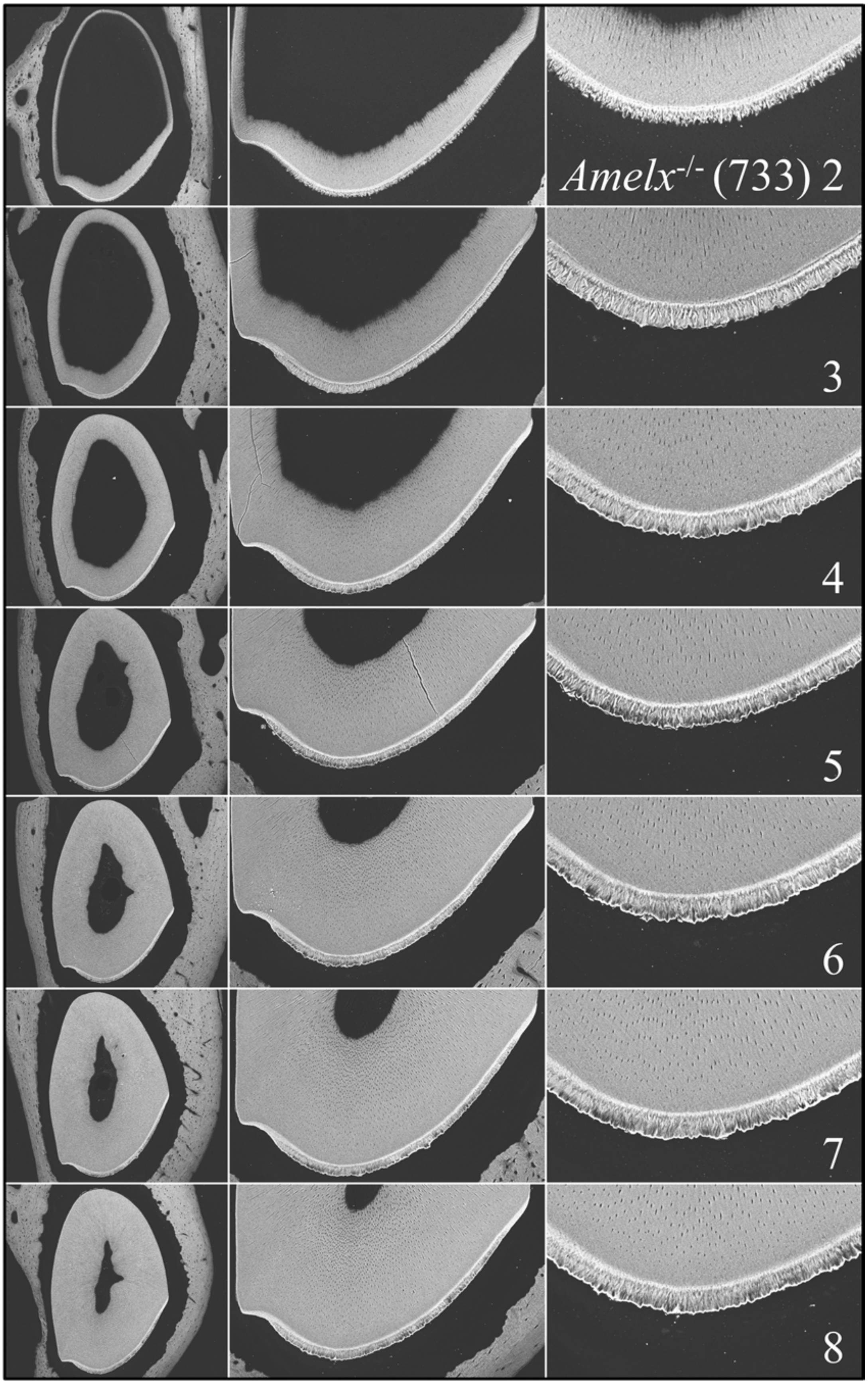

S11 Appendix. Backscatter electron microscopy of successive cross sections from 1 mm increments starting basally (top) and moving incisally (bottom) along the 7-week mandibular incisor of *Amelx*$^{-/-}$ mouse 733.

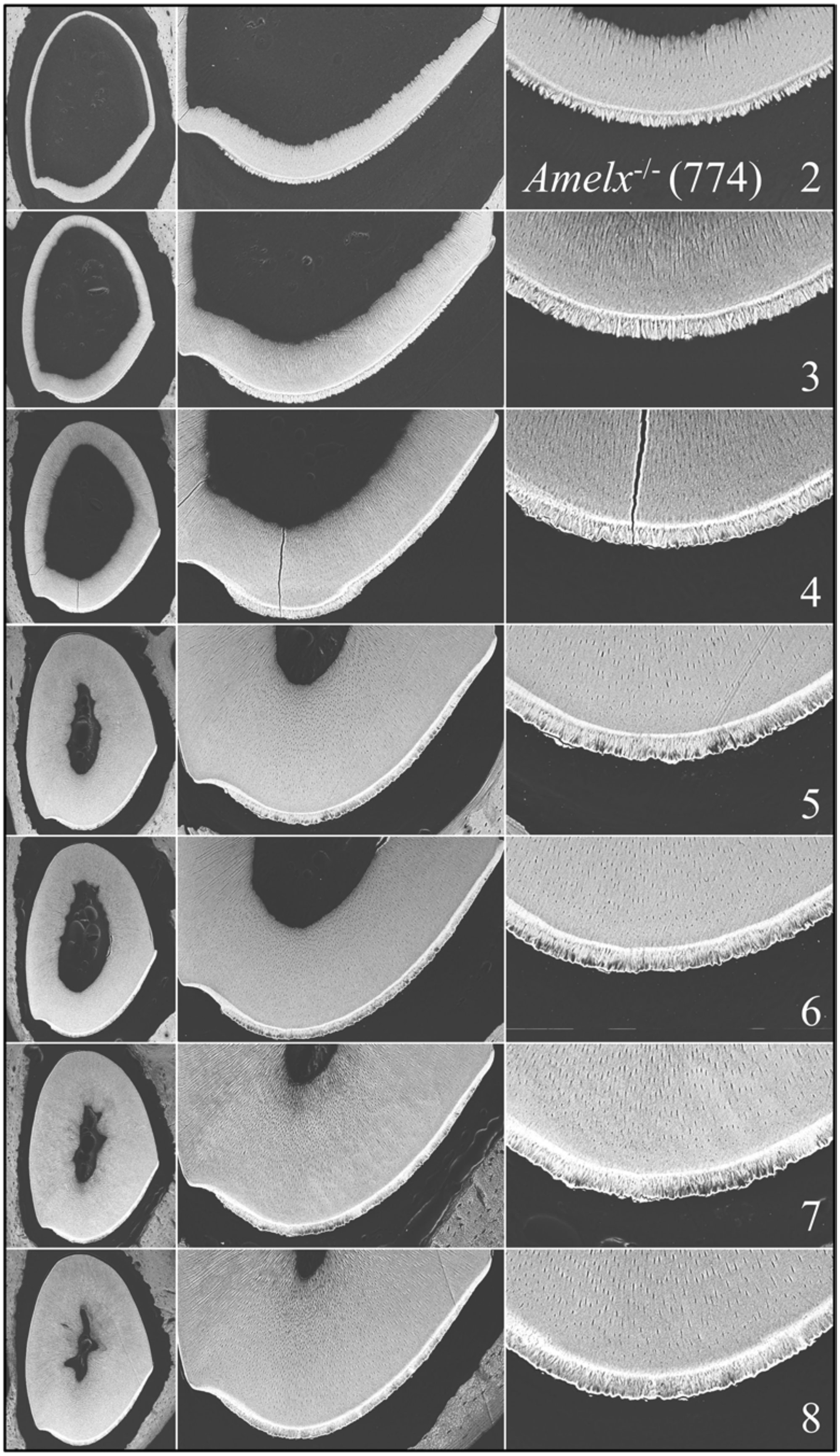

S12 Appendix. Backscatter electron microscopy of successive cross sections from 1 mm increments starting basally (top) and moving incisally (bottom) along the 7-week mandibular incisor of *Amelx*$^{-/-}$ mouse 774.

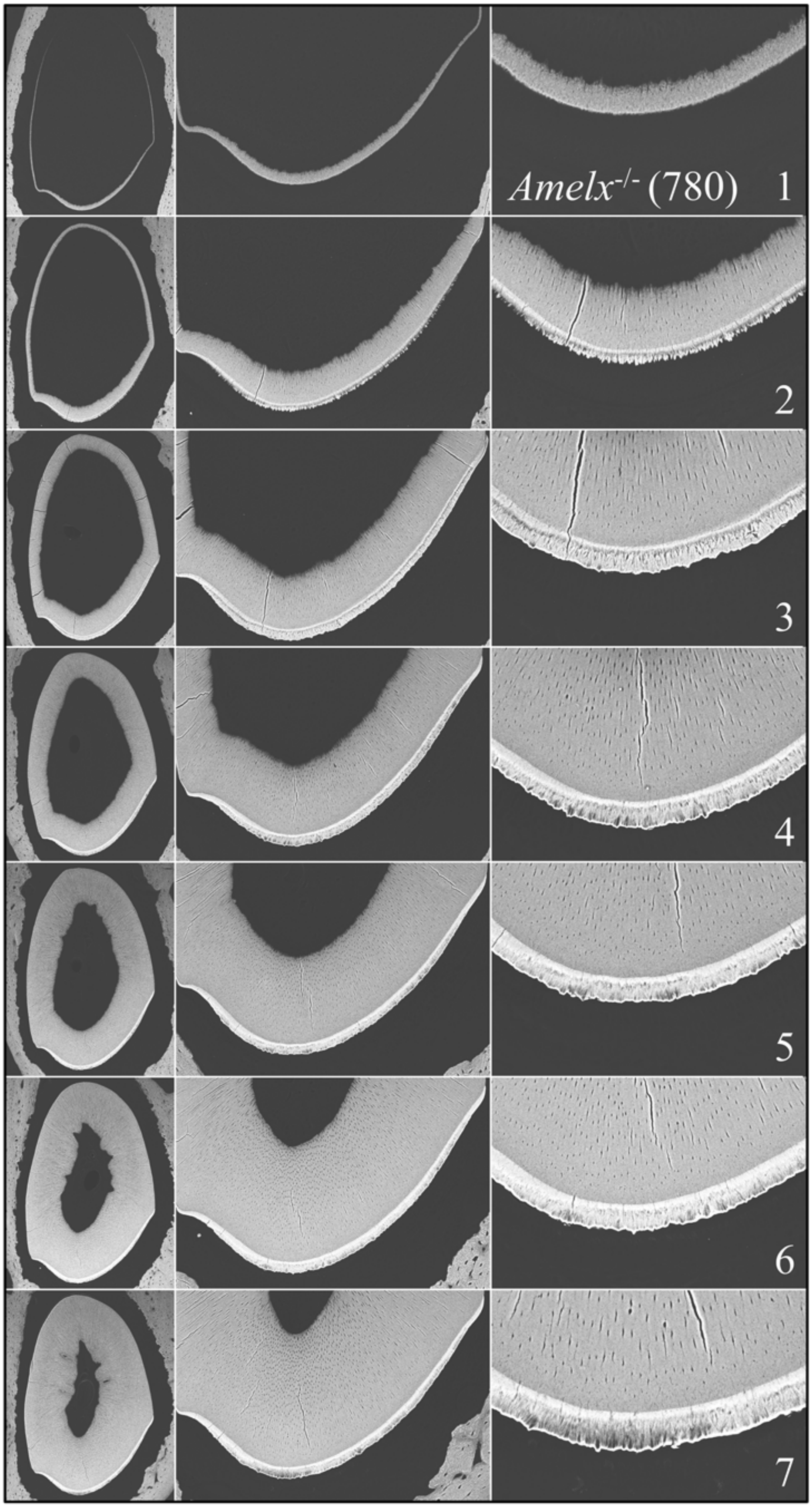

S13 Appendix. Backscatter electron microscopy of successive cross sections from 1 mm increments starting basally (top) and moving incisally (bottom) along the 7-week mandibular incisor of *Amelx*$^{-/-}$ mouse 780.

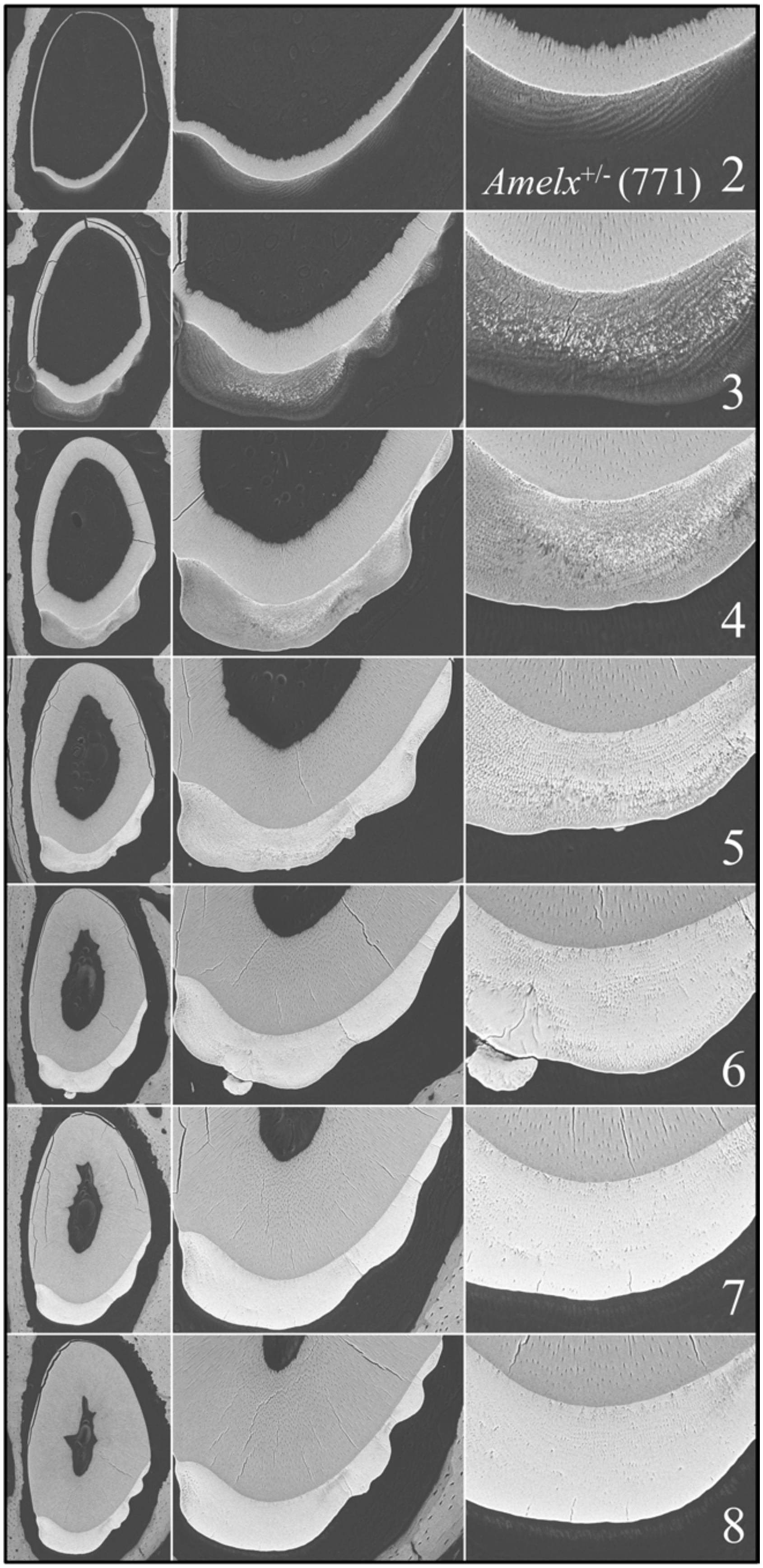

S14 Appendix. Backscatter electron microscopy of successive cross sections from 1 mm increments starting basally (top) and moving incisally (bottom) along the 7-week mandibular incisor of *Amelx*$^{+/-}$ mouse 771.

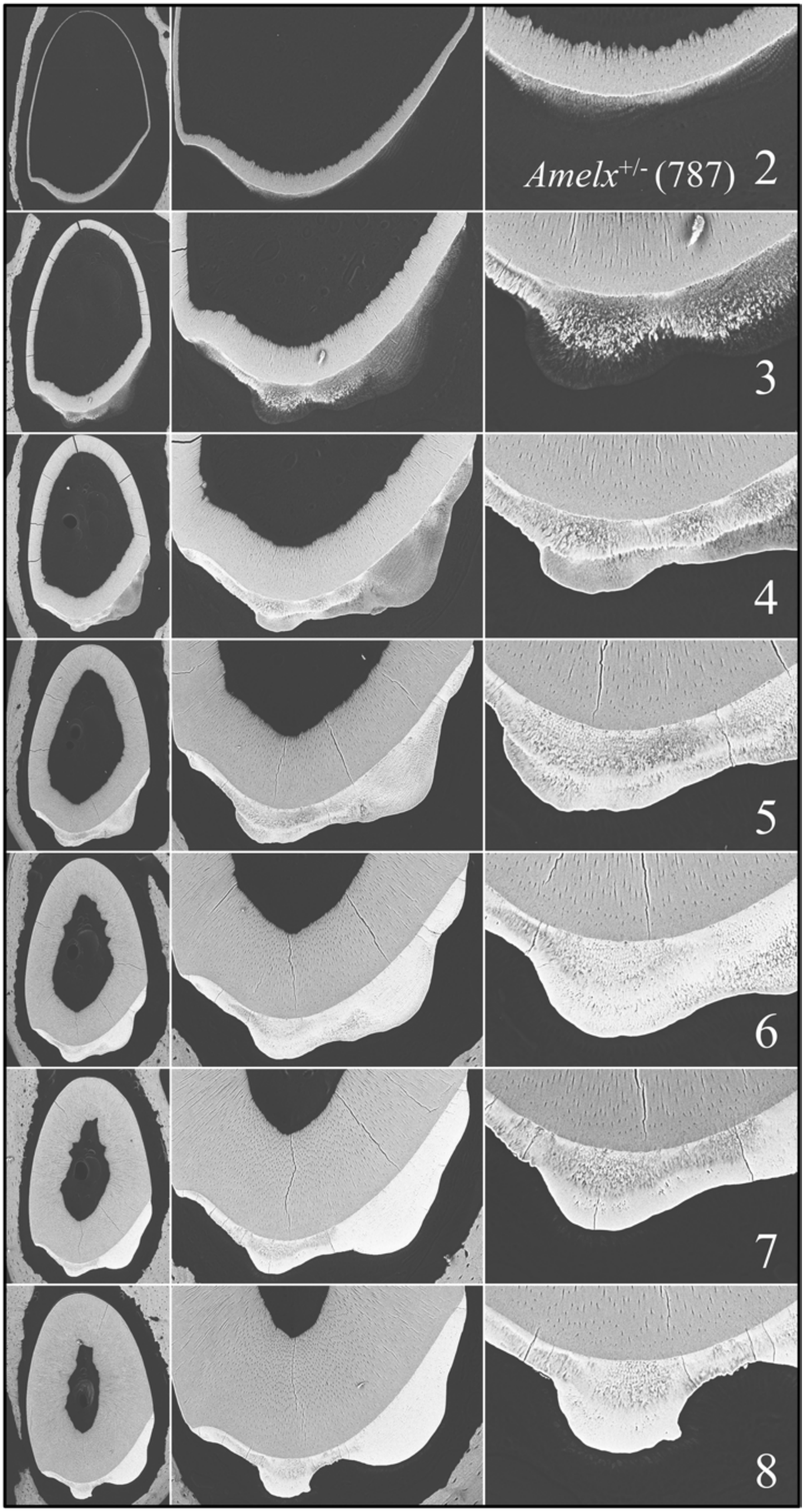

S15 Appendix. Backscatter electron microscopy of successive cross sections from 1 mm increments starting basally (top) and moving incisally (bottom) along the 7-week mandibular incisor of *Amelx*$^{+/-}$ mouse 787.

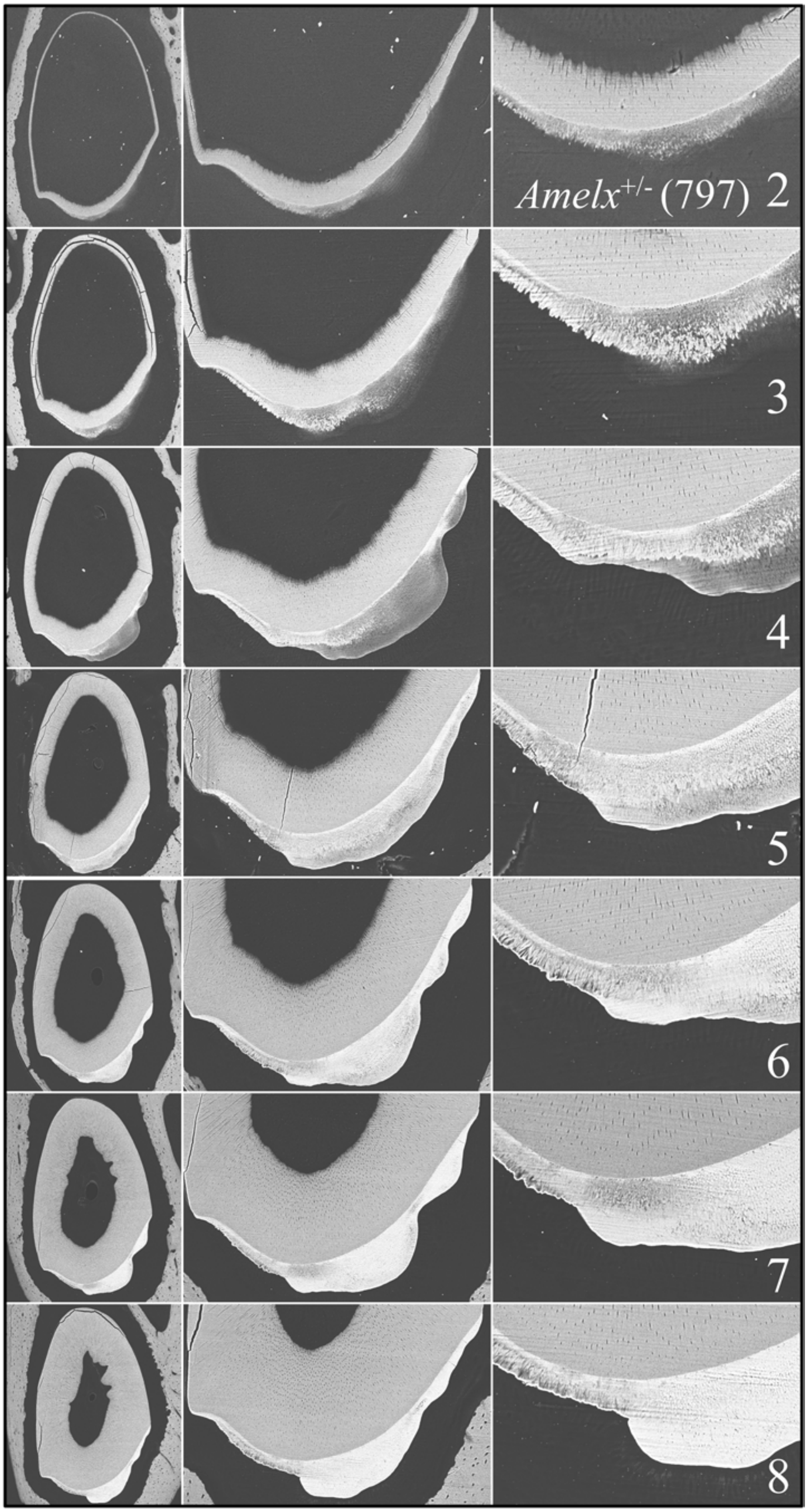

S16 Appendix. Backscatter electron microscopy of successive cross sections from 1 mm increments starting basally (top) and moving incisally (bottom) along the 7-week mandibular incisor of *Amelx*$^{+/-}$ mouse 797.

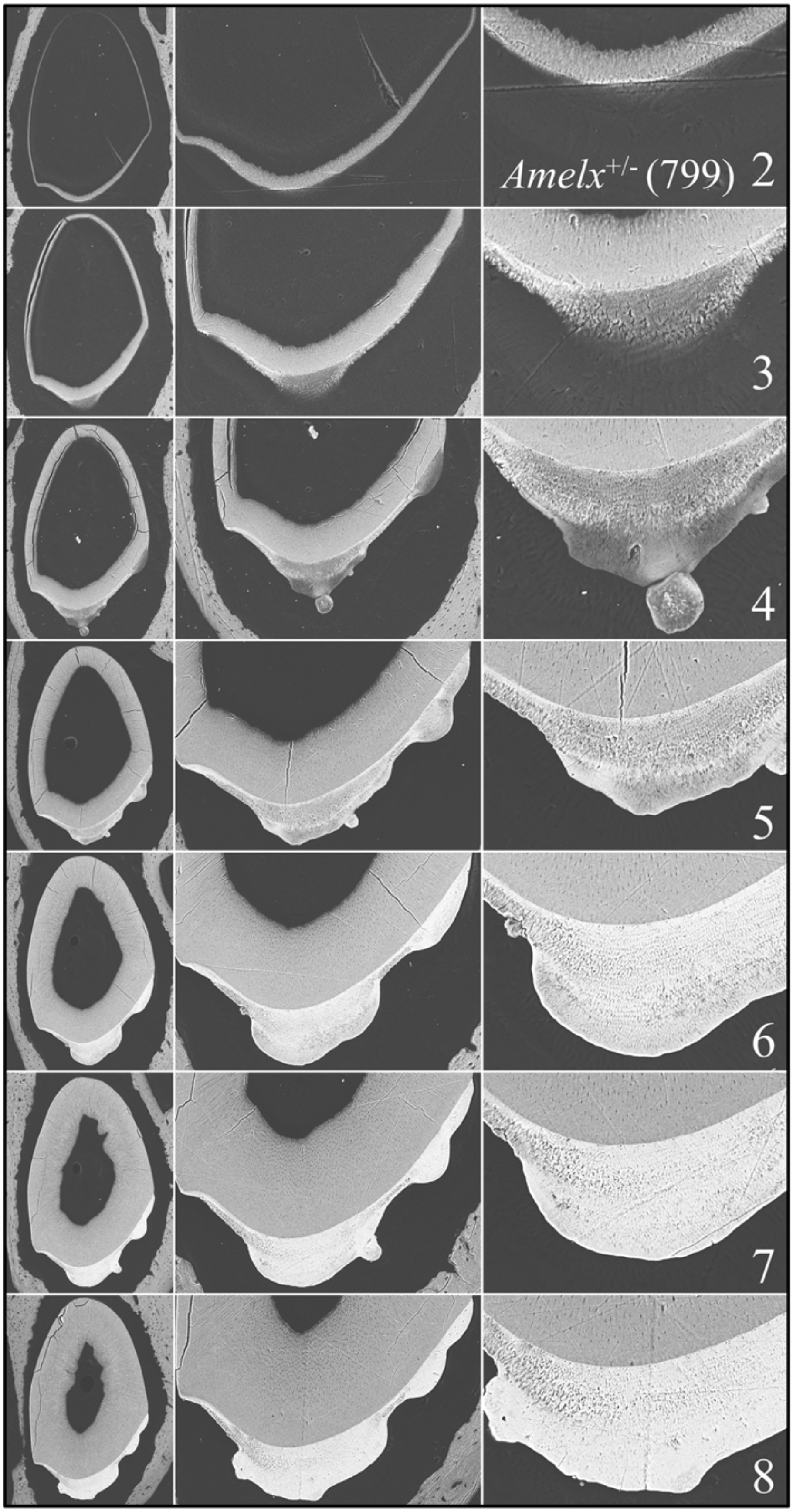

S17 Appendix. Backscatter electron microscopy of successive cross sections from 1 mm increments starting basally (top) and moving incisally (bottom) along the 7-week mandibular incisor of *Amelx*$^{+/-}$ mouse 799.

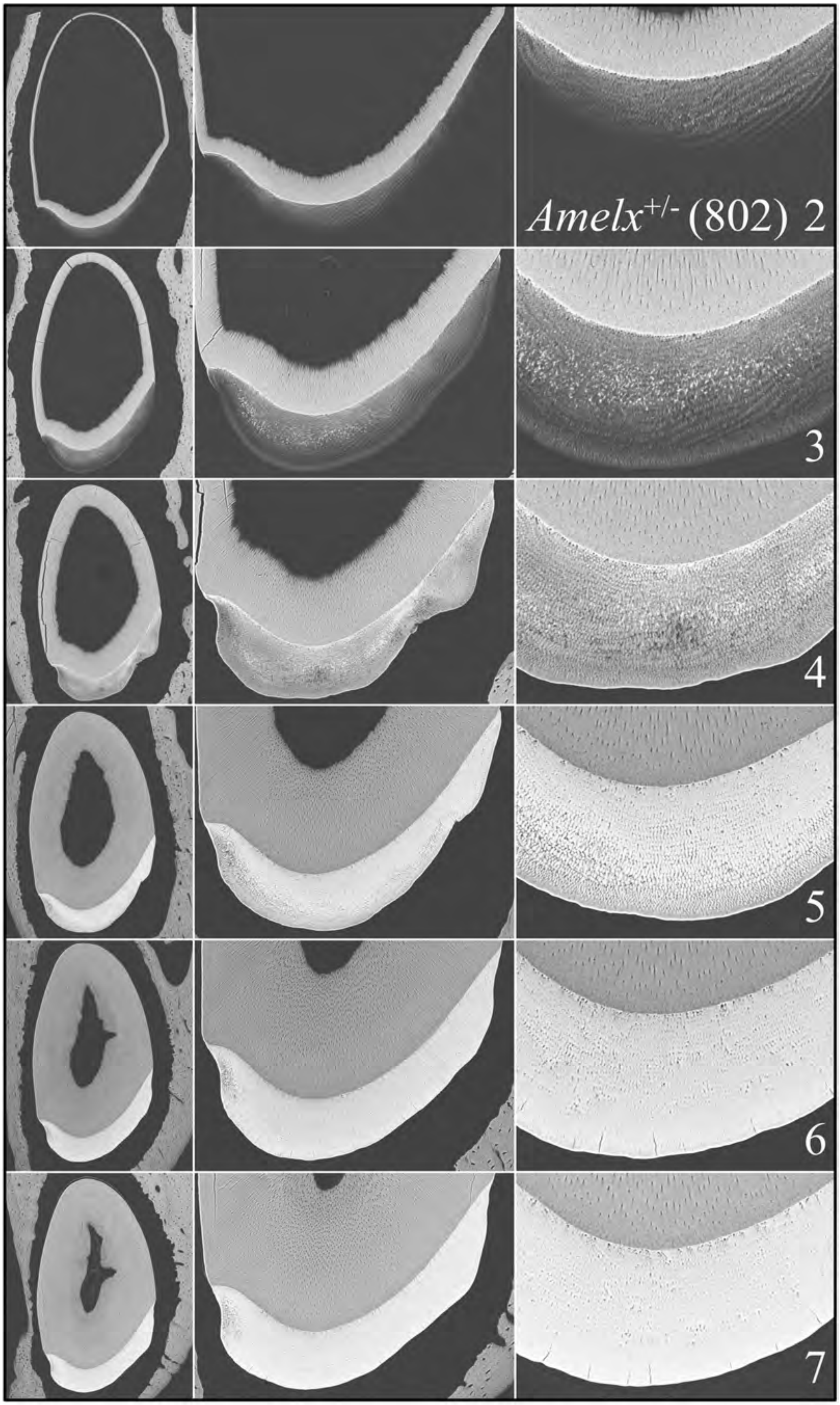

S18 Appendix. Backscatter electron microscopy of successive cross sections from 1 mm increments starting basally (top) and moving incisally (bottom) along the 7-week mandibular incisor of *Amelx*$^{+/-}$ mouse 802.

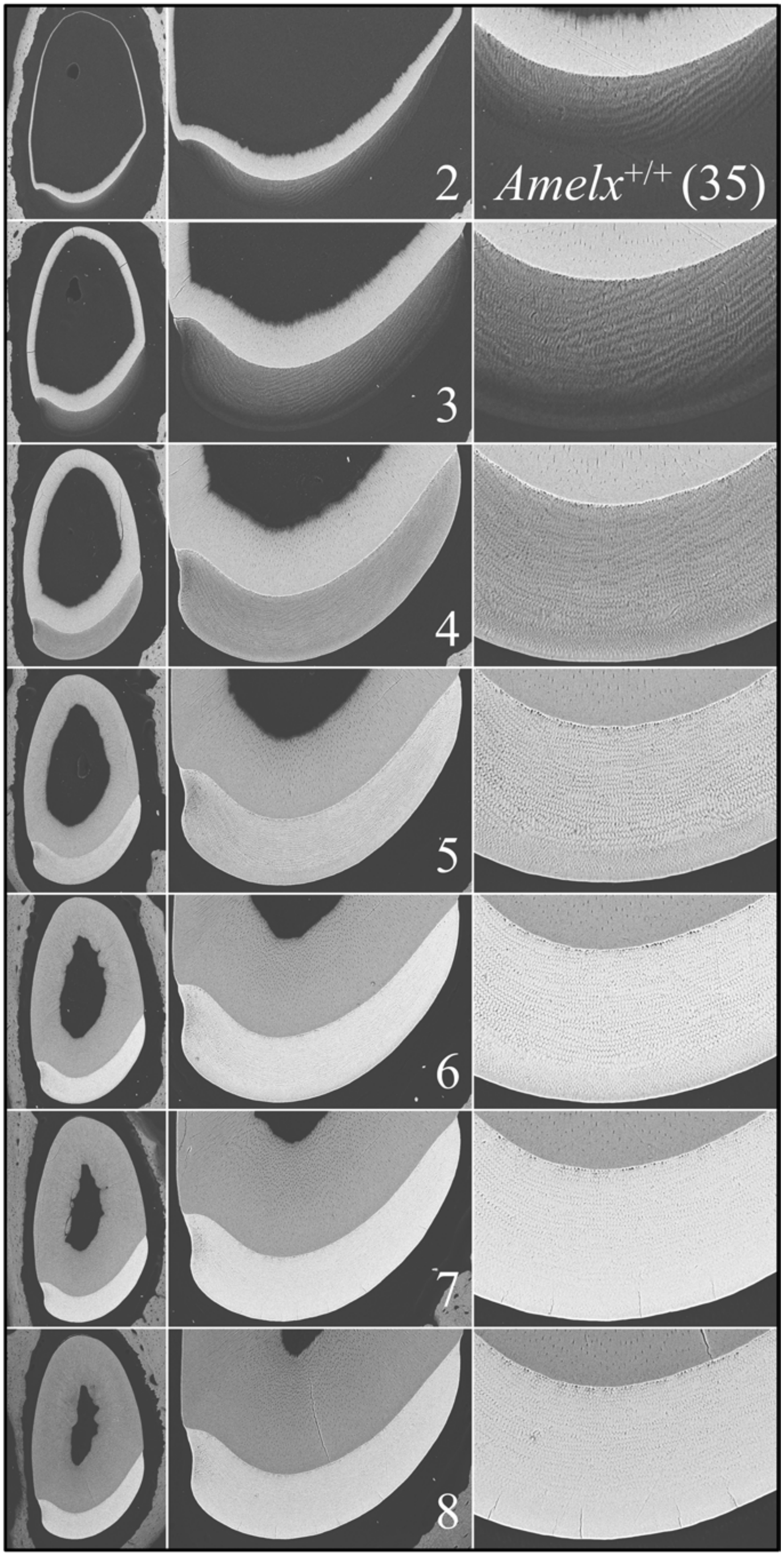

S19 Appendix. Backscatter electron microscopy of successive cross sections from 1 mm increments starting basally (top) and moving incisally (bottom) along the 7-week mandibular incisor of *Amelx*$^{+/+}$ mouse 35.

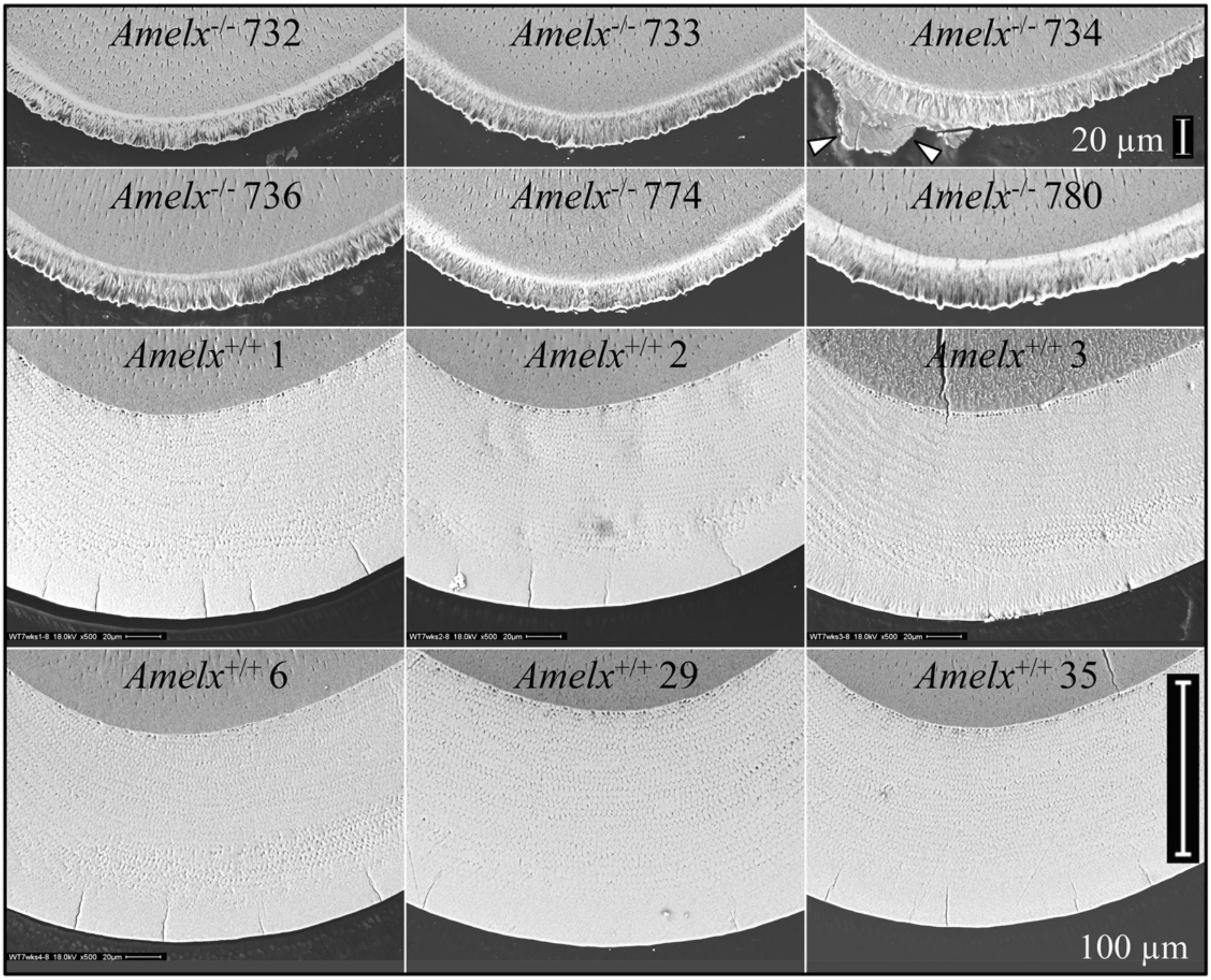

S20 Appendix. Backscatter electron microscopy images for enamel thickness. The enamel thickness at the height of contour for the 6 *Amelx*$^{-/-}$ incisors was 20.3±3.3 µm. The enamel thickness at the the height of contour for the 6 *Amelx*$^{+/+}$ incisors was 122.3±7.9 µm.

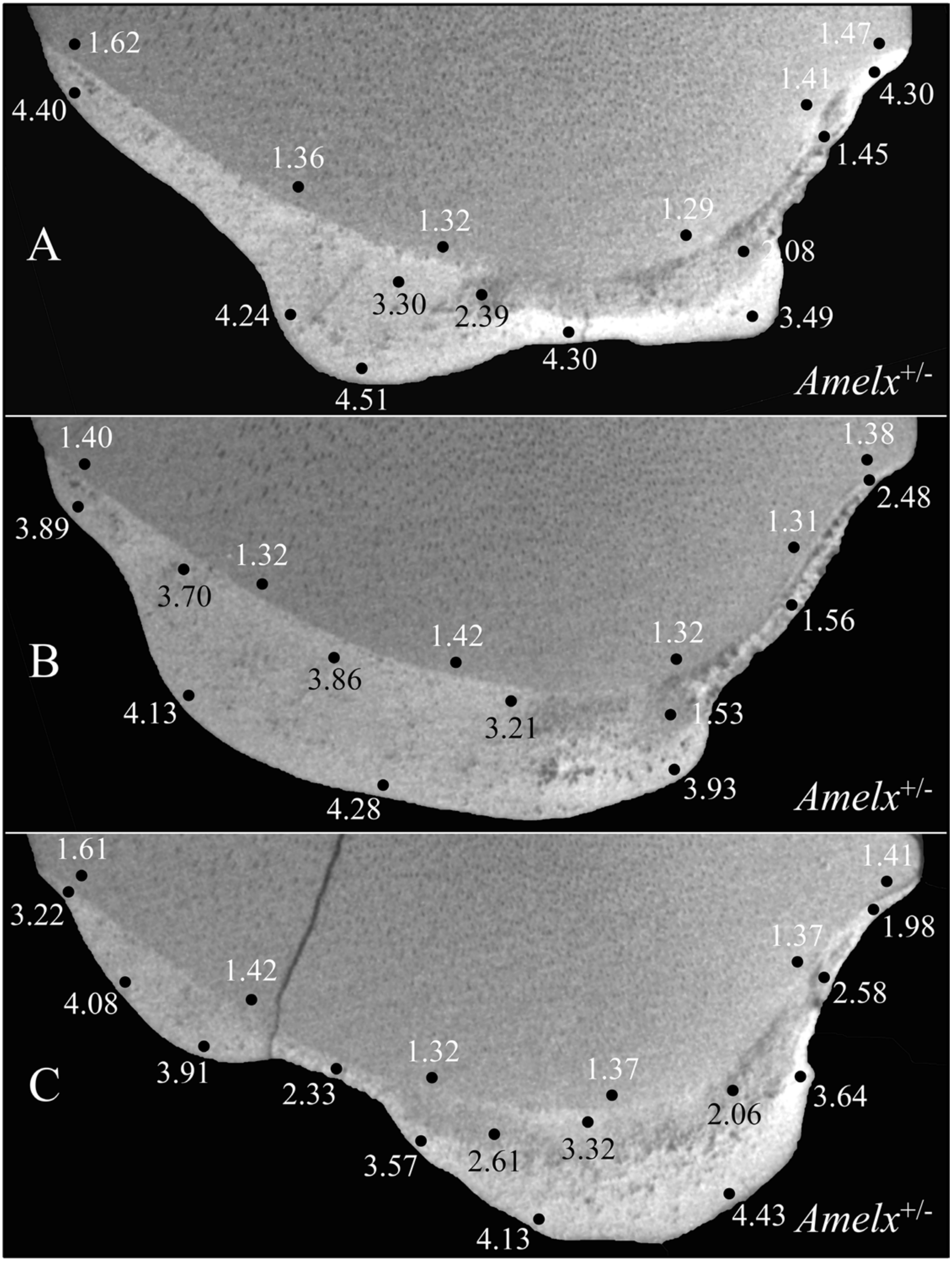

A
1.62
4.40
1.36
1.32
3.30
2.39
4.24
4.51
4.30
1.29
2.08
3.49
1.47
4.30
1.41
1.45
Amelx+/-
B
1.40
3.89
3.70
1.32
3.86
1.42
4.13
3.21
4.28
1.32
1.53
3.93
1.38
2.48
1.31
1.56
Amelx+/-
C
1.61
3.22
4.08
1.42
3.91
2.33
1.32
3.57
2.61
1.37
3.32
4.13
2.06
4.43
1.41
1.98
1.37
2.58
3.64
Amelx+/-

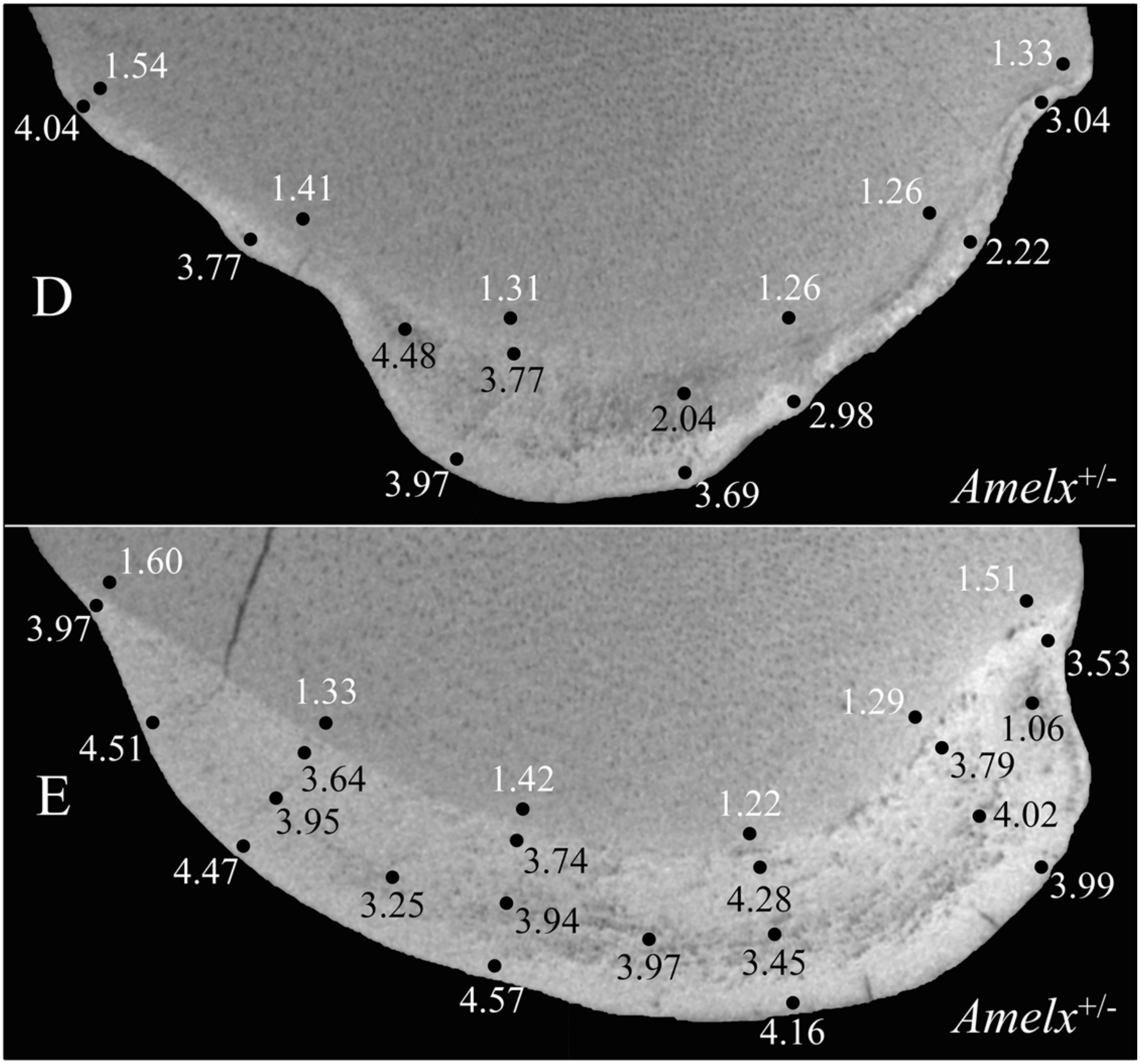

Amelx$^{+/-}$ Dentin total: 1.39±0.10 Gpa

A. 1.41±0.12 B. 1.36±0.05 C. 1.42±0.10 D. 1.35±0.11 E. 1.39±0.14

Amelx$^{+/-}$ Enamel total: 3.46±0.91 Gpa

A. 3.45±1.11 B. 3.26±1.04 C. 3.22±0.83 D. 3.40±0.80 E. 3.81±0.82

S21 Appendix. *Amelx*$^{+/-}$ nanohardness testing. Backscatter electron microscopy images of the 5 *Amelx*$^{+/-}$ mandibular incisor cross-sections used for nanohardness testing and the hardness (Gpa) at each indent site. The average dentin and enamel hardness values for all sites each of the 5 samples (A through E) are shown. The combined average hardness of all of the *Amelx*$^{+/-}$ dentin indents in all 5 samples was 1.39±0.10 Gpa. The combined average hardness of all of the *Amelx*$^{+/-}$ enamel indents in all 5 samples was 3.46±0.91 Gpa.
